# A CuAAC–Hydrazone–CuAAC Trifunctional Scaffold for the Solid-Phase Synthesis of Trimodal Compounds: Possibilities and Limitations

**DOI:** 10.3390/molecules201019310

**Published:** 2015-10-23

**Authors:** Benjamin Fabre, Jan Pícha, Václav Vaněk, Miloš Buděšínský, Jiří Jiráček

**Affiliations:** Institute of Organic Chemistry and Biochemistry, Academy of Sciences of the Czech Republic, v.v.i., Flemingovo n. 2, 16610 Praha 6, Czech Republic; E-Mails: fabre@uochb.cas.cz (B.F.); picha@uochb.cas.cz (J.P.); vasek@uochb.cas.cz (V.V.); budesinsky@uochb.cas.cz (M.B.)

**Keywords:** click chemistry, multifunctional scaffold, solid-phase synthesis, protein mimics, copper, hydrazone, hydrazide

## Abstract

We present a trifunctional scaffold designed for the solid-phase synthesis of trimodal compounds. This scaffold holds two alkyne arms in a free and TIPS-protected form for consecutive CuAAC (copper(I)-catalyzed azide–alkyne cycloaddition), one Fmoc-protected hydrazide arm for reaction with aldehydes, and one carboxylic acid arm with CF_2_ groups for attachment to the resin and ^19^F-NMR quantification. This scaffold was attached to a resin and derivatized with model azides and aliphatic, electron-rich or electron-poor aromatic aldehydes. We identified several limitations of the scaffold caused by the instability of hydrazones in acidic conditions, in the presence of copper during CuAAC, and when copper accumulated in the resin. We successfully overcame these drawbacks by optimizing synthetic conditions for the derivatization of the scaffold with aromatic aldehydes. Overall, the new trifunctional scaffold combines CuAAC and hydrazone chemistries, offering a broader chemical space for the development of bioactive compounds.

## 1. Introduction

**Scheme 1:**
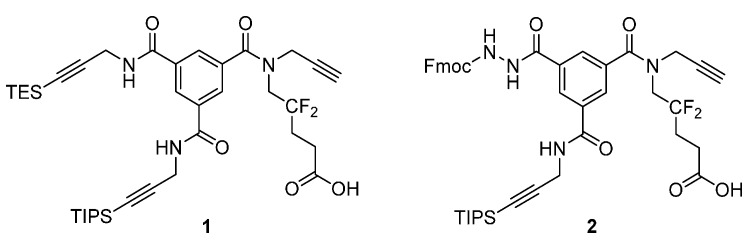
Previously developed scaffold **1** [4] and scaffold **2** (designed in this study).

Multifunctional scaffolds are precious tools used in chemical biology [1] and polymer science [2]. Such scaffolds bear two or more arms, each with orthogonal chemical properties. This allows for the construction of complex multimodal structures by simply “plugging in” different fragments (e.g., fluorescence tags, polymers, drugs) into the scaffold arms [1], which can be used to mimic discontinuous protein epitopes [3]. With this in mind, we developed a trifunctional scaffold specifically designed for the combinatorial solid-phase syntheses of protein binders [4]. We utilized solid-phase synthesis, which is a convenient and widely used method in combinatorial chemistry that generates simple mixtures of compounds by splitting and mixing the resin [5] and allows for clean post-reaction work-up. Scaffold **1** (Scheme 1) comprises three propargylamine arms with free, TES-protected, and TIPS-protected arms to facilitate three successive copper(I)-catalyzed azide–alkyne cycloadditions (CuAACs) with different azides; this strategy was first developed by Aucagne’s group [6]. Compound **1** is assembled on a central rigid core (trimesic acid) that directs the three arms in distinct positions. An additional fluorinated arm enables both the attachment of the scaffold onto the resin and quantitative analyses by ^19^F-NMR [4].

Previously, we found that scaffold **1** could be improved, specifically in the context of solid-phase combinatorial synthesis. First, only a limited number of azides are commercially available, which limits the size of the libraries and thus the chemical variants. Second, we reported [4] that TES groups are unstable in high copper concentrations, which is problematic when using bulky/hindered azides that often require higher copper loads for complete coupling. Thus, we developed a new scaffold by replacing the TES-protected alkyne arm with a hydrazide moiety (compound **2**, Scheme 1). We chose the hydrazide functionality because (i) it can be easily incorporated into the trimesic acid template; (ii) hydrazides can be easily “clicked” onto aldehydes; (iii) a wide variety of aldehydes are commercially available; and (iv) hydrazone and CuAAC chemistries are, in principle, orthogonal. Here, we report the solution synthesis of scaffold **2** and optimization of its solid-phase derivatization with azides and aldehydes. We also discuss the scope and limitations of this new trifunctional scaffold and, in particular, the compatibility of the CuAAC and acylhydrazone chemistries in the solid phase.

## 2. Results and Discussion

### 2.1. Synthesis in Solution: Preparation of Scaffold **2**

**Scheme 2:**
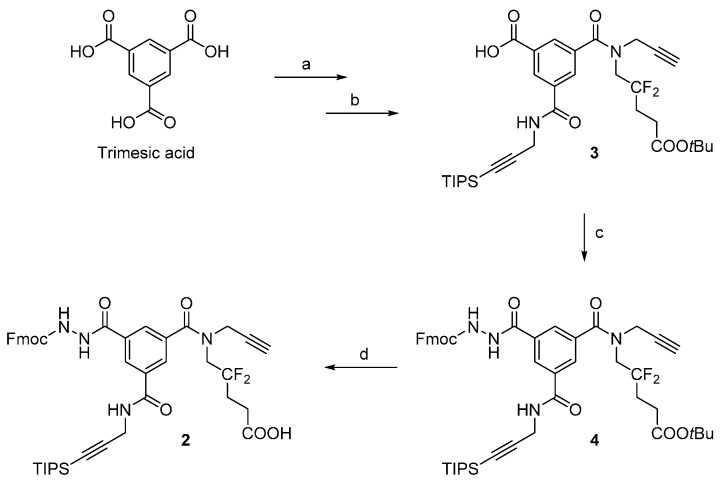
Synthesis of scaffold **2**. Reaction conditions: for the synthesis of **3** (steps (**a**,**b**)), refer to our previous report [4]; (**c**) Fmoc-hydrazine, DIPEA, PyBroP, ACN, 60 °C, 3 h, 57%–67%; (**d**) 50% TFA/DCM (*v*/*v*), r.t., 1 h, 87%–91%.

We synthesized scaffold **2** in solution from diamide **3**, which was prepared as previously described [4] (Scheme 2). During the amide coupling of diamide **3** and Fmoc-hydrazine [7], using conditions used for the synthesis of scaffold **1** (*i.e.*, PyBrop, DMF, and TEA), we observed the partial deprotection of the Fmoc-hydrazide moiety. Consequently, we used ACN to slow the Fmoc deprotection [8] and used the bulkier DIPEA as a base. Heating (60 °C, 3 h) was necessary to generate compound **4**, and a TLC analysis of the reaction mixture showed no dibenzofulvene adduct or Fmoc deprotection. After removal of the *tert*-butyl ester in 50% TFA/DCM (*v*/*v*), we obtained the carboxylic acid **2** in high purity (97%, HPLC chromatogram in Supplementary Figure S4). Similar to scaffold **1**, scaffold **2** presents two alkyne sites in free and TIPS-protected form for CuAAC, an Fmoc-protected hydrazide for the “click-reaction” with aldehydes, and one arm with a carboxylic acid site for attachment to the resin and a CF_2_ group for ^19^F-NMR quantification.

### 2.2. Synthesis on Resin: Low Copper Load

Similar to our previous study [4], we used a PEG-based ChemMatrix resin [9,10] because it swells well in both polar and nonpolar solvents and permits the use of *t*BuOH/H_2_O solvent mixtures for CuAAC. We also used the Ramage linker, which can be cleaved in low TFA concentrations (3%–5%) to afford terminal amides [11].

To attach scaffold **2** to the resin, for the reasons mentioned above, we again used ACN as the solvent and DIPEA as the base. We estimated the loading of scaffold **2** on the resin by determining the quantity of unreacted scaffold **2** present in the washing solutions (by UV spectrometry, as explained in the Experimental Section). To spare scaffold **2** and limit steric hindrance on the resin, we loaded twice as much resin as scaffold **2**, which led to 40%–43% loadings of scaffold **2** in several repeated runs.

**Scheme 3:**
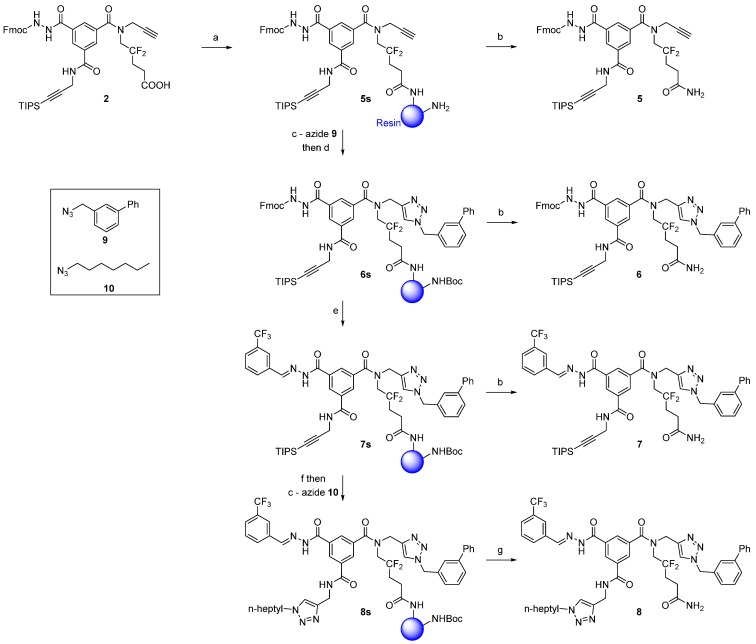
Solid-phase derivatization of scaffold **2**. Reaction conditions: (**a**) Ramage ChemMatrix (2 eq.), PyBroP (2 eq.), DIPEA (1.5 eq.) in ACN, r.t., 6 h; (**b**) 5% TFA/DCM (*v*/*v*), r.t., 2 × 30 min; (**c**) azide **9** or **10** (5 eq.), sodium ascorbate (0.5 eq.), CuSO_4_·5H_2_O (0.1 eq.), tBuOH/water (1:1), r.t., 16 h for azide **9**, 16 h + 4 h for azide **10**; (**d**) Boc_2_O (10 eq.), DIPEA (2 eq.), r.t., 3 h; (**e**) 3-(trifluoromethyl)benzaldehyde (5 eq.), 10% TEA/ACN (*v*/*v*), r.t., 16 h; (**f**) TBAF (5 eq.), DMF, r.t., 3 × 1.5 h; (**g**) 5% TFA/DCM (*v*/*v*), r.t., 2 × 30 min, overall crude yield from **2**: 24% OR; gaseous H_2_S treatment (30 min) followed by 5% TFA/DCM (*v*/*v*), r.t., 2 × 30 min, overall crude yield from **2**: 41%.

As a consequence of this partial loading, free primary amine groups remained on the resin. To inhibit the reaction of these amines with the aldehyde used for acylhydrazone formation, we capped the free amines with Boc-protecting groups (step d, Scheme 3).

The CuAAC on the TIPS-protected alkyne was carried out last because TBAF can cleave Fmoc groups [8,12] and the resulting free hydrazide (R-CO-NH-NH_2_) is unstable (see below). In contrast, the CuAAC on the free alkyne and the acylhydrazone formation should be interchangeable. However, we observed for a model azide that the first CuAAC was significantly faster if performed before the acylhydrazone formation (Scheme S6 and Figure S3).

Following the results from these preliminary experiments, we used the following synthetic order: the first CuAAC on the free alkyne, acylhydrazone formation, and the second CuAAC on the TIPS-protected alkyne. We then synthesized model compound **8** (Scheme 3), and used azides **9** and **10** to determine whether CuAAC with bulky and lipophilic azides reacted on the polar ChemMatrix resin in a *t*BuOH/H_2_O solvent mixture.

The first low-copper-load CuAAC (0.1 equivalent of copper) was completed after 16 h and produced compound **6** in high purity. Then, we used 20% piperidine in DMF to remove the Fmoc group from the resin-bound compound **6s**. However, cleavage from the resin produced a multi-component mixture, which was unexpected because the Fmoc-deprotection of compound **6** in solution rapidly afforded the pure free hydrazide. Performing sequential Fmoc deprotections and acylhydrazone formations from **6s** also afforded multi-component mixtures. We hypothesized that the free hydrazide may have decomposed, and we therefore aimed to trap it with an aldehyde and carry out a one-pot Fmoc-deprotection/acylhydrazone formation. Here, we used triethylamine (TEA) as a base because it deprotects Fmoc groups relatively quickly and does not interfere with the condensation of the free hydrazide with aldehydes. Thus, treating the resin-bound compound **6s** with 3-(trifluoromethyl)benzaldehyde (5 equivalents) in 10% TEA/ACN (*v*/*v*) led to the acylhydrazone **7** in good purity (62%, HPLC chromatogram in Supplementary Figure S5). This one-step Fmoc-deprotection/acylhydrazone formation has the potential to be a useful tool in “click chemistry”, as Fmoc hydrazine can be easily introduced onto carboxylic acids by simple amide coupling and the resulting Fmoc-protected hydrazide can be smoothly converted to acylhydrazone in a single step.

The final product, **8**, was easily obtained by the subsequent removal of the TIPS group with TBAF and a second low-copper-load CuAAC (0.1 equivalent of copper). This second CuAAC was slower than the first and required two treatments (16 h and 4 h) to reach completion, possibly due to the slow diffusion of the highly lipophilic and bulky azide **10** into the polar resin. Crude compound **8** had good HPLC purity (71%, Supplementary Figure S6), and elemental analysis showed an acceptably low amount of copper (100 ppm). ^19^F-NMR analysis [4] was used to determine the quantity of desired compound, and 4.8 mg of crude **8** (theoretically 5.1 μmol) showed 4.8 μmol (94%) of fluorinated compound(s) (Supplementary Figure S11).

In this first experiment, the crude yield of compound **8** was modest (24%) and lower than expected [4]. However, treatment of the resin with hydrogen sulfide prior to the final cleavage significantly improved the yields (see below), and we obtained 39 mg of crude compound **8** (41 μmol, 41%, if pure) by starting from 82 mg of scaffold **2** (100 μmol).

### 2.3. Synthesis on Resin: High Copper Load

Large/bulky azido ligands often require high copper concentrations to react efficiently with alkynes [13]. However, we previously [4] observed that high copper loads led to the deprotection of TES groups, which could be problematic when using scaffold **1** (Scheme 1) in CuAAC on the first unprotected alkyne. In these cases, catalytic amounts of copper could lead to incomplete coupling, whereas higher copper loads could potentially generate double cycloaddition products at the free and TES-protected alkyne arms.

**Scheme 4:**
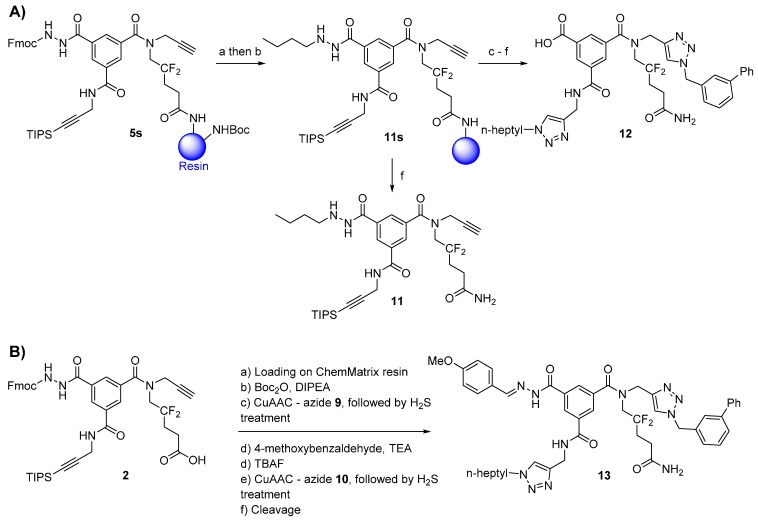
Study of the scope of the reaction. (**A**) Reactions with the aliphatic aldehyde. Reaction conditions: (**a**) 1-butyraldehyde (5 eq.), 10% TEA/ACN (*v*/*v*), r.t., 16 h; (**b**) NaBH_4_ (10 eq.), dry EtOH, r.t., 3 h; (**c**) azide **9** (5 eq.), sodium ascorbate (0.5 eq.), CuSO_4_·5H_2_O (0.1 eq.), *t*BuOH/water (1:1), r.t., 16 h; (**d**) TBAF (5 equiv.), DMF, r.t., 3 × 1.5 h; (**e**) azide **10** (5 eq.), sodium ascorbate (0.5 eq.), CuSO_4_·5H_2_O (0.1 eq.), *t*BuOH/water (1:1), r.t., 16 h + 4 h; (**f**) 5% TFA/DCM (*v*/*v*), r.t., 2 × 30 min; (**B**) Reactions with the aromatic aldehyde. The reaction conditions are identical to those in Scheme 3 (steps **a**, **c**–**f**), apart from the final cleavage from the resin: 5% TFA/DCM (*v*/*v*), 2% TIS (*v*/*v*), r.t., 2 × 30 min, overall crude yield from **2**: 43%.

In order to verify whether our new scaffold tolerated high copper loads during both CuAACs, we re-synthesized compound **8** (Scheme 3) using one equivalent of copper sulfate in each CuAAC. However, after the final cleavage from the resin, we obtained a mixture containing compound **8** and carboxylic acid **12** (structure in Scheme 4A) in a 1:1 ratio (Figure S7A).

Because the only difference between the two syntheses of **8** was the high copper load, we suspected that residual copper ions retained in the PEG-resin participated in the degradation of **8**. Previously, we reported that treatment of the final compounds with gaseous hydrogen sulfide led to the formation of insoluble copper salts (presumably CuS) that could be easily filtered out [4]. To investigate whether treating the resin-bound compound directly with hydrogen sulfide could prevent copper ions from interacting with the target compounds and to understand when the acylhydrazone hydrolysis takes place, we repeated the synthesis of compound **8** using low (0.1 equivalent) and high (1 equivalent) loads of copper sulfate in each CuAAC. We then treated or did not treat the resin with hydrogen sulfide after each CuAAC and analyzed the intermediate compounds **6** and **7** by HPLC. While high copper concentrations were problematic, we were unable to distinguish whether the copper ions participated only in acylhydrazone degradation during cleavage from the resin or if they also interfered with acylhydrazone formation. However, we observed that the presence of copper (II) ions in solution has a negligible impact on the condensation reaction between benzhydrazide and 3-(trifluoromethyl)benzaldehyde (Supplementary Materials, Figure S2 and Table S1). In either case, hydrogen sulfide efficiently “inactivated” the copper ions present in the resin. Thus, synthesis using one equivalent of copper and treatment of the resin with hydrogen sulfide after each CuAAC generated compound **8** in purity comparable to synthesis with a low copper load (64% purity, Supplementary Figure S7B). Furthermore, compound **8** was obtained in a higher crude yield (47%) and elemental analysis verified that the content of copper in the final crude compound **8** was low (15 ppm) despite the use of a higher copper load.

### 2.4. Synthesis on Resin: Scope and Limitations

The scope of the CuAAC [14,15], the condensation between hydrazines and aldehydes, the Boc-protection of amines, and the deprotection of TIPS group by TBAF are well studied and compatible with a wide range of functional groups. The main limitations of our chemistry stem from acidic cleavage (5% TFA/DCM) that precludes the presence of acid-labile groups (e.g., acetal) and the use of hydrogen sulfide gas. Hydrogen sulfide in solution is a reducing agent that reduces azide functions in water [16], and is also a nucleophilic species that reacts with disulfides [17].

Additionally, we studied whether different aldehydes are compatible with the presented chemistry. We chose 1-butyraldehyde and 4-methoxybenzaldehyde as models of aliphatic and electron-rich aldehydes, respectively (Scheme 4).

#### 2.4.1. Aliphatic Aldehydes

First, we observed that aliphatic acylhydrazone was unstable in the acidic cleavage mixture (5% TFA/DCM) and quickly hydrolyzed. We attempted to solve this problem by reducing the acylhydrazone group to the hydrazide, synthesizing the substituted hydrazide **11s** from the resin-bound scaffold **5s** (Scheme 4A). The Fmoc-protected hydrazide **5s** was reacted with 1-butyraldehyde (low copper load), and the resulting acylhydrazone was reduced to the substituted hydrazide **11s** directly on the resin with sodium borohydride because milder reducing agents (*i.e.*, sodium triacetoxyborohydride and sodium cyanoborohydride) were inefficient. Upon cleavage from the resin, we obtained the substituted hydrazide **11** in good purity (70% by HPLC, Supplementary Figure S8). It was important that the acylhydrazide formation was performed before the first CuAAC, as the presence of residual copper in the resin results in a messy reduction (appearance of a black colloid when sodium borohydride is added) [18].

The synthesis was completed following the protocol described for compound **8** under low copper loads. However, after final cleavage from the resin, the substituted hydrazide moiety was fully hydrolyzed and we obtained only the benzoic acid derivative **12** (Supplementary Figure S9). Because compound **11** was stable in acidic conditions, it appears that copper ions participate in the hydrolysis, supporting previous reports that Cu(II) ions catalyze the apparent hydrolysis of poly-substituted aliphatic hydrazides [19,20,21] by initiating aerobic oxidation of the hydrazide moiety [21]. Hence, we verified that Cu(II) ions indeed promote the hydrolysis of *N*′-butylbenzohydrazide in aqueous solution (Supporting Information, Figure S1). Treating the resin with gaseous hydrogen sulfide after each CuAAC did not lessen this effect, and the substituted aliphatic acylhydrazide was still hydrolyzed.

#### 2.4.2. Electron-Rich Aromatic Aldehydes

Next, we investigated the synthetic compatibility of the electron-rich 4-methoxybenzaldehyde. Electron-rich aromatic aldehydes are less electrophilic than aliphatic or electron-poor aromatic aldehydes and react more slowly with nucleophiles. Compared to the low copper load protocol shown in Scheme 3, we added triisopropylsilane (TIS) to scavenge the carbocations formed in the acidic medium, as these carbocations may add to the electron-rich anisole ring (Scheme 4B). After each CuAAC, the resin was treated with gaseous H_2_S, and compound **13** was obtained as the major product but in lower purity levels than compound **8** (HPLC chromatogram in Supplementary Figure S10). The lower purity of crude **13** prompted us to investigate its stability in an acidic non-aqueous solvent (5% TFA/DCM, *i.e.*, cleavage mixture), an acidic aqueous solvent (50% ACN/water + 0.1% TFA, *i.e.*, HPLC solvent), and the HPLC solvent with Cu(II) ions (one equivalent of copper sulfate pentahydrate). These experiments revealed that 1 h treatment in 5% TFA/DCM did not affect compound **13**, whereas the two aqueous conditions decomposed **13** to generate different hydrolysis products (see discussion in Supporting Materials, Table S2 and Scheme S5).

### 2.5. Final Remarks

We report here observations that could be useful to chemists combining the CuAAC and acylhydrazone chemistry or solid-phase CuAAC.

First, the aliphatic acylhydrazide **11** was hydrolyzed to the carboxylic acid derivative during the synthesis. Using a model aliphatic acylhydrazide (Supporting Materials, Figure S1), we observed that such hydrolysis occurs rapidly in aqueous solution with Cu(II) ions (CuSO_4_·5H_2_O) and more slowly in the CuAAC conditions used here (CuSO_4_·5H_2_O and sodium ascorbate). Thus, CuAAC in aqueous medium should not be performed in the presence of an aliphatic acylhydrazide moiety as the acylhydrazide could be hydrolyzed.

Second, the presence of copper ions (presumably trapped in the PEG-resin) affected hydrazone reduction and promoted the hydrolysis of aromatic acylhydrazone during the cleavage from the resin, hindering solid-phase synthesis. Here, we demonstrated that treatment of the resin with gaseous hydrogen sulfide during the synthesis efficiently inactivated the copper ions, which could potentially inactivate other interfering metal ions [22].

## 3. Experimental Section

### 3.1. General Information

Unless otherwise stated, reagents and solvents used in this study were obtained from commercial suppliers and used without purification. EtOH was dried with 3-Å molecular sieves, as described in the literature [23]. The solvents were evaporated at 55 °C and 2 kPa, and the products were dried over phosphorus pentoxide at r. t. and 13 Pa. TLC were run on aluminum plates coated with silica gel (60F_254_, Merck, Darmstadt, Germany). The compounds were visualized by exposure to UV light at 254 nm. Flash chromatography purifications were carried out on silica gel (40–63 μm, Fluka, Darmstadt, Germany). Analytical RP-HPLC chromatography of the compounds were carried out on a Waters HPLC system (Waters 1525 Binary HPLC Pump and Waters 2787 Dual λ Absorbance Detector, Milford, MA, USA) using Nucleosil 120-5 C8 column (250 × 4.6 mm, 5 μm, Watrex, Praha, Czech Republic) at a flow rate of 1 mL/min. Solvent A: 0.1% TFA (*v*/*v*) in H_2_O. Solvent B: 0.1% TFA (*v*/*v*) in CH_3_CN. The following gradients were used: t = 0 min/50% B, t = 30 min/100% B, t = 31 min/50% B (Method A), or t = 0 min/20% B, t = 30 min/100% B, t = 31 min/20% B (Method B). The compounds were detected at 218 and 254 nm. The purity of a given compound was determined by integration of the whole spectra at 218 nm. Preparative RP-HPLC chromatography of compounds was carried on the same Waters HPLC system but using a Vydac 214TP101522 C4 column (250 × 22 mm, 10–15 μm, Columbia, MD, USA) at a flow rate of 9 mL/min. The gradients and the detection were the same as used in analytical HPLC. HRMS spectra were obtained on an LTQ-orbitrap XL FTMS mass spectrometer (Thermo Fisher, Bremen, Germany) in electrospray ionization mode or in case HRMS (EI) on GCT Premier (Waters). ^1^H- and ^13^C-NMR spectra were measured on a Bruker AVANCE-600 instrument (^1^H at 600 MHz and ^13^C at 150.9 MHz frequency, Rheinstatten, Germany) or AVANCE-500 instrument (^1^H- at 500 MHz and ^13^C- at 125.8 MHz frequency, Rheinstatten, Germany) in DMSO-*d*_6_. The 2D-H,H-COSY, 2D-H,C-HSQC and 2D-H,C-HMBC spectra were recorded and used for the structural assignment of proton and carbon signals. The presence of amide bonds and bulky arms leads to a slow rotation of the trimesic acid substituents and results in line broadening of many signals. The increasing temperature leads to the narrowing of signals or to the coalescence of doubled peaks but some signals could not be detected even at 100 °C (in particular signals from the tertiary amide arm). The ^19^F-NMR spectrum was measured on a Bruker AVANCE-500 instrument (Rheinstatten, Germany) at 470.4 MHz. For the quantitative estimation of the fluorine containing compounds the ^19^F proton-decoupled spectra with suppression of heteronuclear NOE during relaxation delay (3 s) were measured. A known amount of BrCF_2_COOEt was added to the measured solution and used as a standard for quantitative analysis. IR spectra were recorded on Bruker IFS 55 Equinox apparatus (Rheinstatten, Germany). UV-Vis spectrometry was carried out on a Perkin Elmer Lambda 25 system (Norwalk, CT, USA).

### 3.2. Syntheses

#### 3.2.1. Compound **4**

A suspension of compound **3** [4] (5.6 g, 8.8 mmol), PyBrop (6.2 g, 13.3 mmol, 1.5 eq.), Fmoc-hydrazine [7] (6.7 g, 26.3 mmol, 3.0 eq.) and DIPEA (3.08 mL, 17.7 mmol, 2.0 eq.) in ACN (150 mL) was stirred at 60 °C. After 3 h, the reaction was cooled to r.t. and then filtered to remove excess Fmoc-hydrazine. The filtrate was evaporated, suspended in EtOAc (60 mL), and filtered again to remove the remaining Fmoc-hydrazine. The EtOAc filtrate was poured into a separatory funnel, washed a saturated NH_4_Cl solution (2 × 30 mL) and brine (50 mL), dried over anhydrous sodium sulfate, filtered, and evaporated to give 8.5 g of brown oil. The crude product was purified by flash chromatography on silica (200 g of silica, elution with a linear gradient—5% to 65%—of EtOAc in toluene), resulting in compound **4** as a pale yellow solid (4.5 g, 58%, 57%–67% in repeated runs; R*_f_* = 0.3 in 7:3 toluene/EtOAc).

Analytical HPLC: (Method A) *t*_R_ = 24.5 min. ^1^H-NMR (500 MHz; DMSO-*d*_6_; T = 100 °C): δ_H_ 1.06 (21H, m, Si(CH(CH_3_)_2_)_3_), 1.41 (9H, s, C(CH_3_)_3_), 2.22 (2H, m, –CH_2_–), 2.38 (2H, t, ^3^*J*_HH_ = 7.5 Hz, –CH_2_–CO), 3.12 (1H, t, ^4^*J*_HH_ = 2.4 Hz, –C≡CH), 4.03 (2H, t, ^3^*J*_HF_ = 14.2 Hz, –CH_2_–N), 4.18 (2H, d, ^3^*J*_HH_ = 5.6 Hz, –CH_2_–N), 4.25 (2H, d, ^4^*J*_HH_ = 2.4 Hz, –CH_2_–N), 4.27 (1H, t, ^3^*J*_HH_ = 7.0 Hz, >CH– Fmoc), 4.40 (2H, d, ^3^*J*_HH_ = 7.0 Hz, –CH_2_–O Fmoc), 7.30 (2H, t, ^3^*J*_HH_ = 7.5 Hz, 2 × CH_arom_), 7.40 (2H, t, ^3^*J*_HH_ = 7.5 Hz, 2 × CH_arom_), 7.70 (2H, d, ^3^*J*_HH_ = 7.5 Hz, 2 × CH_arom_), 7.84 (2H, d, ^3^*J*_HH_ = 7.5 Hz, 2 × CH_arom_), 8.04 (1H, t, ^3^*J*_HH_ = 1.5 Hz, CH_arom_), 8.05 (1H, t, ^3^*J*_HH_ = 1.5 Hz, CH_arom_), 8.45 (1H, t, ^3^*J*_HH_ = 1.5 Hz, CH_arom_), 8.87 (1H, t, ^3^*J*_HH_ = 5.6 Hz, >NH). ^13^C-NMR (125.8 MHz; DMSO-*d*_6_; T = 100 °C): δ_C_ 11.04 (3C, Si(CH(CH_3_)_2_)_3_)), 18.56 (6C, Si(CH(CH_3_)_2_)_3_), 27.99 (3C, –C(CH_3_)_3_), 28.22 (1C, –CH_2_–), 29.94 (1C, –CH_2_–N), 30.26 (1C, t, ^2^*J*_CF_ = 24.4 Hz, –CH_2_–), 39.40 (1C, –CH_2_–N), 47.05 (1C, >CH– Fmoc), 49.45 (1C, –CH_2_–N), 66.75 (1C, –CH_2_–O Fmoc), 75.72 (1C, ≡C–H), 78.60 (1C, ≡C–), 80.40 (1C, C(CH_3_)_3_), 82.36 (1C, ≡C–), 105.64 (1C, ≡C–), 120.13 (2C, 2 × CH_arom_), 123.47 (1C, t, ^1^*J*_CF_ = 244.5 Hz, >CF_2_), 125.34 (2C, 2 × CH_arom_), 127.20 (2C, 2 × CH_arom_), 127.79 (2C, 2 × CH_arom_), 128.19 (1C, CH_arom_), 128.21 (1C, CH_arom_), 128.56 (1C, CH_arom_), 133.68 (1C, C_arom_), 135.38 (1C, C_arom_), 135.88 (1C, C_arom_), 141.02 (2C, C_arom_), 143.94 (2C, C_arom_), 156.32 (1C, –CO–NH), 164.96 (1C, –CO–NH), 170.39 (1C, –CO–NH), 170.74 (1C, –CO–O) (–CO–N< missing). IR (KBr) ν_max_ = 3420 (m, vbr, NH), 3306 (m, C≡C-H), 2944 (s, CH_3_ TIPS), 2177 (w, C≡C), 1731, 1657, 1526 (s, br, C=O). HRMS (*m/z*): calcd for C_48_H_58_F_2_N_4_NaO_7_Si^+^ [M + Na]^+^ 891.39350, found 891.39383.

#### 3.2.2. Scaffold **2**

TFA (10 mL) was added to a solution of **4** (4.4 g, 5.1 mmol) in DCM (10 mL) at 0 °C. The reaction mixture was allowed to reach r.t. and was stirred for 1 h. The reaction mixture was co-evaporated with toluene to afford 5.6 g of brownish foam. The crude product was purified by flash chromatography on silica (200 g of silica, elution with a linear gradient—0% to 60% over 40 min—of 1% AcOH/EtOAc (*v*/*v*) in toluene), resulting in compound **2** as a beige solid (3.6 g, 87%, 87%–91% in repeated runs; R*_f_* = 0.3 in 1% AcOH/EtOAc (*v*/*v*) in toluene 1:1).

Analytical HPLC: (Method A) *t*_R_ = 17.9 min. ^1^H-NMR (500 MHz; DMSO-*d*_6_; T = 100 °C): δ_H_ 1.06 (21H, m, Si(CH(CH_3_)_2_)_3_), 2.24 (2H, m, –CH_2_–), 2.42 (2H, t, ^3^*J*_HH_ = 7.5 Hz, –CH_2_–CO), 3.12 (1H, t, ^4^*J*_HH_ = 2.4 Hz, –C≡CH), 4.04 (2H, t, ^3^*J*_HF_ = 14.5 Hz, –CH_2_–N), 4.18 (2H, d, ^3^*J*_HH_ = 5.6 Hz, –CH_2_–N), 4.25 (2H, d, ^4^*J*_HH_ = 2.4 Hz, –CH_2_–N), 4.27 (1H, t, ^3^*J*_HH_ = 7.0 Hz, >CH– Fmoc), 4.40 (2H, d, ^3^*J*_HH_ = 7.0 Hz, –CH_2_–O Fmoc), 7.30 (2H, t, ^3^*J*_HH_ = 7.5 Hz, 2 × CH_arom_), 7.40 (2H, t, ^3^*J*_HH_ = 7.5 Hz, 2 × CH_arom_), 7.71 (2H, d, ^3^*J*_HH_ = 7.5 Hz, 2 × CH_arom_), 7.84 (2H, d, ^3^*J*_HH_ = 7.5 Hz, 2 × CH_arom_), 8.04 (1H, t, ^3^*J*_HH_ = 1.5 Hz, CH_arom_), 8.06 (1H, t, ^3^*J*_HH_ = 1.5 Hz, CH_arom_), 8.44 (1H, t, ^3^*J*_HH_ = 1.5 Hz, CH_arom_), 8.88 (1H, t, ^3^*J*_HH_ = 5.6 Hz, >NH). ^13^C-NMR (125.8 MHz; DMSO-*d_6_*; T = 100 °C): δ_C_ 10.50 (3C, Si(CH(CH_3_)_2_)_3_)), 18.03 (6C, Si(CH(CH_3_)_2_)_3_), 26.21 (1C, t, ^3^*J*_CF_ = 5.2 Hz, –CH_2_–), 29.40 (1C, –CH_2_–N), 29.74 (1C, t, ^2^*J*_CF_ = 24.2 Hz, –CH_2_–), 38.81 (1C, –CH_2_–N), 46.52 (1C, >CH– Fmoc), 66.21 (1C, –CH_2_–O Fmoc), 75.21 (1C, ≡C–H), 78.10 (1C, ≡C–), 81.83 (1C, ≡C–), 105.12 (1C, ≡C–), 119.60 (2C, 2 × CH_arom_), 124.00 (1C, t, ^1^*J*_CF_ = 244.5 Hz, >CF_2_), 124.81 (2C, 2 × CH_arom_), 126.66 (2C, 2 × CH_arom_), 127.25 (2C, 2x CH_arom_), 127.64 (1C, CH_arom_), 127.68 (1C, CH_arom_), 128.02 (1C, CH_arom_), 133.14 (1C, C_arom_), 134.84 (1C, C_arom_), 135.35 (1C, C_arom_), 140.49 (2C, 2 × C_arom_), 143.41 (2C, 2 × C_arom_), 155.79 (1C, –CO–NH), 164.43 (1C, –CO–NH), 169.86 (1C, –CO–NH), 172.19 (1C, –COOH) (–CO–N< and one –CH_2_–N missing). IR (KBr) ν_max_ = 3294 (m, br, C≡C-H and NH), 2944 (s, CH_3_ TIPS), 2178 (w, C≡C), 1723, 1657, 1528 (s, br, C=O). HRMS (*m*/*z*): calcd for C_44_H_49_F_2_N_4_O_7_Si^−^ [M – H]^−^ 811.33441, found 811.33420.

#### 3.2.3. Compound **5**

Ramage ChemMatrix^®^ resin (667 mg, 200 μmol, 0.3 mmol/g, Aldrich 727792) with free amino groups was swelled in MeOH, DCM, and ACN (10 minutes each, using 10 mL of solvent/g of resin) in a fritted polypropylene syringe. The resin was then washed with 3 × 5 mL ACN (1 min each). A solution of scaffold **2** (82 mg, 100 μmol), PyBroP (93 mg, 200 μmol, 2.0 eq.), and DIPEA (26 μL, 150 μmol, 1.5 eq.) in ACN (2.5 mL) was added to the resin. The resulting mixture was stirred at r.t. for 6 h. The resin was washed as described for the determination of the loading of compound **2** (vide-infra) and with 3 × 5 mL of MeOH and DCM (1 min each) to afford resin-bound compound **5s**.

A small sample of resin-bound compound **5s** (*ca.* 10% of the total amount) was transferred to a fritted glass micro-reactor and dried under reduced pressure. The product was cleaved from the resin with a 5% TFA/DCM (*v*/*v*) solution (5 mL) for 30 minutes. The resin was subsequently slowly washed with 3 × 5 mL of 5% TFA/DCM. All solutions were combined and evaporated to dryness. It is recommended that the cleavage procedure be repeated until a constant weight of the dried compound is reached. Typically, two TFA cleavage procedures were sufficient to recover all of the material from the resin. Compound **5** (12 mg) was purified by RP-HPLC (Method A) and lyophilized (5.1 mg, white powder).

Analytical HPLC: (Method A) *t*_R_ = 14.1 min. ^1^H-NMR (500 MHz; DMSO-*d*_6_; T = 100 °C): δ_H_ 1.05 (21H, m, Si(CH(CH_3_)_2_)_3_), 2.19 (2H, m, –CH_2_–), 2.28 (2H, br t, ^3^*J*_HH_ = 7.5 Hz, –CH_2_–CO), 3.11 (1H, t, ^4^*J*_HH_ = 2.4 Hz, –C≡CH), 4.02 (2H, t, ^3^*J*_HF_ = 14.5 Hz, –CH_2_–N), 4.18 (2H, d, ^3^*J*_HH_ = 5.5 Hz, –CH_2_–N), 4.24 (2H, br d, ^4^*J*_HH_ = 2.4 Hz, –CH_2_–N), 4.26 (1H, t, ^3^*J*_HH_ = 6.8 Hz, >CH– Fmoc), 4.40 (2H, d, ^3^*J*_HH_ = 6.8 Hz, –CH_2_–O Fmoc), 7.30 (2H, t, ^3^*J*_HH_ = 7.5 Hz, 2 × CH_arom_), 7.40 (2H, t, ^3^*J*_HH_ = 7.5 Hz, 2 × CH_arom_), 7.70 (2H, d, ^3^*J*_HH_ = 7.5 Hz, 2 × CH_arom_), 7.84 (2H, d, ^3^*J*_HH_ = 7.5 Hz, 2 × CH_arom_), 8.02 (1H, t, ^3^*J*_HH_ = 1.5 Hz, CH_arom_), 8.04 (1H, t, ^3^*J*_HH_ = 1.5 Hz, CH_arom_), 8.42 (1H, t, ^3^*J*_HH_ = 1.5 Hz, CH_arom_), 8.88 (1H, br t, ^3^*J*_HH_ = 5.6 Hz, >NH), 9.08 (1H, br, >NH), 10.33 (1H, br, >NH). ^13^C-NMR (125.8 MHz; DMSO-*d*_6_; T = 100 °C): δ_C_ 10.55 (3C, Si(CH(CH_3_)_2_)_3_)), 18.09 (6C, Si(CH(CH_3_)_2_)_3_), 27.14 (1C, –CH_2_–), 29.47 (1C, –CH_2_–N), 30.04 (1C, t, ^2^*J*_CF_ = 24.6 Hz, –CH_2_–), 38.75 (1C, –CH_2_–N), 46.56 (1C, >CH– Fmoc), 48.81 (1C, –CH_2_–N), 66.28 (1C, –CH_2_–O Fmoc), 75.32 (1C, ≡C–H), 78.15 (1C, ≡C–), 81.91 (1C, ≡C–), 105.15 (1C, ≡C–), 119.67 (2C, 2 × CH_arom_), 123.30 (1C, t, ^1^*J*_CF_ = 244.5 Hz, >CF_2_), 124.87 (2C, 2 × CH_arom_), 126.74 (2C, 2 × CH_arom_), 127.34 (2C, 2 × CH_arom_), 127.72 (2C, 2 × CH_arom_), 128.09 (1C, CH_arom_), 133.19 (1C, C_arom_), 134.90 (1C, C_arom_), 135.46 (1C, C_arom_), 140.54 (2C, 2 × C_arom_), 143.45 (2C, 2 × C_arom_), 155.87 (1C, –CO–NH), 164.54 (1C, –CO–NH), 169.90 (1C, –CO–NH), 172.26 (1C, –CO–NH_2_) (–CO–N< missing). HRMS (*m*/*z*): calcd for C_44_H_51_F_2_N_5_NaO_6_Si^+^ [M + Na]^+^ 834.34689, found 834.34718.

#### 3.2.4. Compound **6**

The resin **5s** was washed with 3 × 5 mL of MeOH and *t*BuOH/water (1:1, *v*/*v*) (1 min each). The following solutions were sequentially added to the resin: (i) azide **9** (105 mg, 500 μmol, 5.0 eq., refer to the Supporting Information for the synthesis) in 1 mL of *t*BuOH/water (1:1, *v*/*v*); (ii) sodium ascorbate (100 μL of a freshly prepared 0.5 M aqueous solution, 50 μmol, 0.5 eq.) in 1 mL of *t*BuOH/water (1:1, *v*/*v*); and (iii) copper(II) sulfate pentahydrate (100 μL of a freshly prepared 0.1 M aqueous solution, 10 μmol, 0.1 eq.) in 1 mL of *t*-BuOH/water (1:1, *v*/*v*). After stirring for 16 h at r.t., the coupling was complete. The resin was washed with 3 × 5 mL of *t*BuOH/water (1:1, *v*/*v*), DMF, DCM, MeOH and DCM (1 min each) to afford the resin-bound compound **6s**.

A small sample of resin-bound compound **6s** (*ca.* 10% of the total amount) was transferred to a fritted glass micro-reactor and dried under reduced pressure. The product was cleaved using the procedure described for compound **5** to give crude compound **6** (*ca.* 18 mg). Compound **6** was purified by RP-HPLC (Method A) and lyophilized (7.3 mg, white powder).

Analytical HPLC: (Method A) *t*_R_ = 17.4 min. ^1^H-NMR (500 MHz; DMSO-*d*_6_; T = 100 °C): δ_H_ 1.05 (21H, m, Si(CH(CH_3_)_2_)_3_), 2.18 (2H, m, –CH_2_–), 2.26 (2H, m, –CH_2_–CO), 3.93 (2H, t, *J*_HF_ = 14.3 Hz, –CH_2_–N), 4.17 (2H, d, ^3^*J*_HH_ = 5.6 Hz, –CH_2_–N), 4.26 (1H, t, ^3^*J*_HH_ = 7.0 Hz, >CH– Fmoc), 4.39 (2H, d, ^3^*J*_HH_ = 7.0 Hz, –CH_2_–O Fmoc), 4.70 (2H, s, –CH_2_–N), 5.62 (2H, s, –CH_2_–N), 7.24 (1H, d, ^3^*J*_HH_ = 7.5 Hz, CH_arom_), 7.29 (2H, t, ^3^*J*_HH_ = 7.5 Hz, 2 × CH_arom_), 7.35 (1H, tt, ^3^*J*_HH_ = 7.5 Hz, ^4^*J*_HH_ = 1.2 Hz, CH_arom_), 7.39 (2H, t, ^3^*J*_HH_ = 7.5 Hz, 2 × CH_arom_), 7.44 (3H, t, ^3^*J*_HH_ = 7.5 Hz, 3 × CH_arom_), 7.59 (4H, m, 4 × CH_arom_), 7.695 (2H, d, ^3^*J*_HH_ = 7.5 Hz, 2 × CH_arom_), 7.84 (2H, d, ^3^*J*_HH_ = 7.5 Hz, 2 × CH_arom_), 8.00 (1H, br s, CH_arom_), 8.04 (1H, br s, CH_arom_), 8.05 (1H, br s, CH_arom_), 8.40 (1H, br s, CH_arom_), 8.86 (1H, t, ^3^*J*_HH_ = 5.6 Hz, >NH), 9.10 (1H, br, >NH), 10.32 (1H, br, >NH). ^13^C-NMR (125.8 MHz; DMSO-*d*_6_; T = 100 °C): δ_C_ 10.50 (3C, Si(CH(CH_3_)_2_)_3_)), 18.04 (6C, Si(CH(CH_3_)_2_)_3_), 27.22 (1C, –CH_2_–), 29.41 (1C, –CH_2_–N), 30.09 (1C, –CH_2_–), 43.89 (1C, –CH_2_–N), 45.51 (1C, >CH– Fmoc), 48.56 (1C, –CH_2_–N), 52.75 (1C, –CH_2_–N), 66.22 (1C, –CH_2_–O Fmoc), 81.83 (1C, ≡C–), 105.12 (1C, ≡C–), 119.61 (2C, CH_arom_), 123.42 (1C, CH_arom_), 124.82 (2C, 2 × CH_arom_), 126.01 (1C, CH_arom_), 126.10 (1C, CH_arom_), 126.34 (2C, 2 × CH_arom_), 126.46 (1C, CH_arom_), 126.67 (2C, 2 × CH_arom_), 127.20 (1C, CH_arom_), 127.27 (2C, 2 × CH_arom_), 127.38 (1C, CH_arom_), 127.82 (1C, CH_arom_), 128.26 (1C, CH_arom_), 128.48 (2C, 2 × CH_arom_), 128.97 (1C, CH_arom_), 134.76 (1C, C_arom_), 136.01 (1C, C_arom_), 136.10 (1C, C_arom_), 139.52 (1C, C_arom_), 140.49 (2C, 2 × C_arom_), 140.55 (1C, C_arom_), 143.40 (2C, 2 × C_arom_), 155.82 (1C, –CO–NH), 164.55 (1C, –CO–NH), 170.18 (1C, –CO–NH), 172.01 (1C, –CO–NH_2_) (–CO–N< and two C_arom_ missing). HRMS (*m*/*z*): calcd for C_57_H_62_F_2_N_8_NaO_6_Si^+^ [M + Na]^+^ 1043.44219, found 1043.44263.

#### 3.2.5. Compound **7**

(1) Capping: The resin **6s** was washed with 3 × 5 mL of DCM (1 min each). A cooled solution (0 °C) of Boc_2_O (*ca*. 220 mg, *ca.* 1 mmol, *ca.* 10 eq.) and DIPEA (35 μL, 200 μmol, 2.0 eq.) in DCM (4 mL) was added to the resin. The resin was left upside down (to avoid projection) at r.t. for 3 h. The resin was washed with 3 × 5 mL of DCM, DMF, DCM and MeOH (1 min each). A Kaiser’s test performed on a few grains of resin confirmed complete capping (negative test).
(2) One-step Fmoc-deprotection/hydrazone formation: The Boc-capped resin was washed with 3 × 5 mL of DCM and ACN (1 min each). A solution of 3-(trifluoromethyl)benzaldehyde (67 μL, 500 μmol, 5.0 eq.) in 10% TEA/ACN (*v*/*v*, 2.5 mL) was added to the resin. The mixture was stirred at r.t. for 16 h. The resin was then washed with 3 × 5 mL of ACN, DMF, DCM, MeOH, and DCM (1 min each) to afford resin-bound compound **7s**.

A small sample of resin-bound compound **7s** (*ca*. 30% of the total amount) was transferred to a fritted glass micro-reactor and dried under reduced pressure. The product was cleaved using the procedure described for compound **5** to give 24 mg of yellow oil. Compound **7** partially hydrolyzed in the HPLC buffer (0.1% TFA, pH ≈ 2) and was thus purified as follows. First, the low-polarity impurities were extracted from the crude compound **7** with 2 min of sonication in 10 mL of Et_2_O. After centrifugation (7200 rpm, 5 min at 20 °C), the supernatant was discarded. The procedure was repeated once, and the final residue was dried to give 16 mg of a pale-yellow solid. Then, the crude product was passed through a C-4 cartridge to remove salts (particularly any remaining copper). Briefly, the crude compound was dissolved in a 30% aqueous AcOH solution (2 mg/mL solution) and loaded onto an activated C-4 cartridge (Chromabond C-4, 3 mL/500 mg). The cartridge was then washed with a 10% aqueous AcOH solution (3 × 2 mL) and water (3 × 2 mL). The product was then eluted with ACN (5 × 2 mL) and lyophilized to give **7** (10 mg, white powder). The purity of crude compound **7** was verified by RP-HPLC (Method A, Supplementary Figure S5). This crude compound was directly used for NMR and MS analyses.

Analytical HPLC: (Method A) *t*_R_ = 17.7 min. ^1^H-NMR (500 MHz; DMSO-*d*_6_; T = 100 °C): δ_H_ 1.05 (21H, m, Si(CH(CH_3_)_2_)_3_), 2.18 (2H, m, –CH_2_–), 2.26 (2H, m, –CH_2_–CO), 3.94 (2H, t, *J*_HF_ = 14.2 Hz, –CH_2_–N), 4.18 (2H, d, ^3^*J*_HH_ = 5.6 Hz, –CH_2_–N), 4.72 (2H, s, –CH_2_–N), 5.64 (2H, s, –CH_2_–N), 7.25 (1H, d, ^3^*J*_HH_ = 7.5 Hz, CH_arom_), 7.35 (1H, tt, ^3^*J*_HH_ = 7.5 Hz, ^4^*J*_HH_ = 1.2 Hz, CH_arom_), 7.44 (3H, t, ^3^*J*_HH_ = 7.5 Hz, 3 × CH_arom_), 7.59 (4H, m, 4 × CH_arom_), 7.68 (1H, t, ^3^*J*_HH_ = 7.6 Hz, CH_arom_), 7.76 (1H, d, ^3^*J*_HH_ = 7.6 Hz, CH_arom_), 7.99 (1H, d, ^3^*J*_HH_ = 7.6 Hz, CH_arom_), 8.02 (2H, br s, 2 × CH_arom_), 8.07 (1H, br s, CH_arom_), 8.11 (1H, br s, CH_arom_), 8.45 (1H, t, ^3^*J*_HH_ = 1.6 Hz, CH_arom_), 8.56 (1H, s, –N=CH), 8.91 (1H, t, ^3^*J*_HH_ = 5.6 Hz, >NH), 11.89 (1H, br s, >NH) (one CH_2_–N missing). ^13^C-NMR (125.8 MHz; DMSO-*d*_6_; T = 100 °C): δ_C_ 10.49 (3C, Si(CH(CH_3_)_2_)_3_)), 18.03 (6C, Si(CH(CH_3_)_2_)_3_), 27.10 (1C, –CH_2_–), 29.40 (1C, –CH_2_–N), 30.08 (1C, –CH_2_–), 44.00 (1C, –CH_2_–N), 52.76 (1C, –CH_2_–N), 81.82 (1C, ≡C–), 105.11 (1C, ≡C–), 122.82 (1C, CH_arom_), 123.46 (1C, CH_arom_), 126.01 (1C, CH_arom_), 126.09 (1C, CH_arom_), 126.32 (3C, 3 × CH_arom_), 126.43 (1C, CH_arom_), 127.18 (1C, CH_arom_), 127.74 (1C, CH_arom_), 128.11 (1C, CH_arom_), 128.47 (2C, 2 × CH_arom_), 128.96 (1C, CH_arom_), 129.57 (1C, CH_arom_), 130.56 (1C, CH_arom_), 139.50 (2C, 2 × C_arom_), 140.55 (1C, C_arom_), 164.56 (1C, –CO–NH), 170.20 (1C, –CO–NH) (–CO–N<, –CO–NH and eight C_arom_ missing). HRMS (*m*/*z*): calcd for C_50_H_56_F_5_N_8_O_4_Si^+^ [M + H]^+^ 955.41085, found 955.41159.

#### 3.2.6 Compound **8** (Low Copper Load)

(1) TIPS deprotection: The resin **7s** was washed with 3 × 5 mL of DMF (1 min each). A solution of TBAF (500 μL of a 1 M solution in DMF, 500 μmol, 5.0 eq.) in DMF (2 mL) was then added to the resin. Three treatments at r.t. for 1.5 h each were necessary to completely remove the TIPS group. Between each treatment, the resin was washed with 3 × 5 mL of DMF (1 min each). The resin was finally washed with 3 × 5 mL of DMF, glacial AcOH, water, DMF, DCM, MeOH, and DCM (1 min each).
(2) Final CuAAC: The resin (terminal-alkyne-bound compound) was washed with 3×5 mL of MeOH and *t*BuOH/water (1:1, *v*/*v*) (1 min each). The following solutions were sequentially added to the resin: (i) 1-azidoheptane **10** (71 mg, 500 μmol, 5.0 eq., refer to Supplementary Materials for the synthesis) in 1 mL of *t*BuOH/water (1:1, *v*/*v*); (ii) sodium ascorbate (100 μL of a freshly prepared 0.5 M aqueous solution, 50 μmol, 0.5 eq.) in 1 mL of *t*BuOH/water (1:1, *v*/*v*); and (iii) copper(II) sulfate pentahydrate (100 μL of a freshly prepared 0.1 M aqueous solution, 10 μmol, 0.1 eq.) in 1 mL of *t*-BuOH/water (1:1, *v*/*v*). Two couplings of 16 h and 4 h at r.t. were necessary for the reaction to reach completion. The resin was washed with 3 × 5 mL of *t*BuOH/water (1:1, *v*/*v*), DMF, DCM, MeOH and DCM (1 min each) to afford resin-bound compound **8s**.

Compound **8** was also prepared as described above without partial TFA cleavages to determine the yield of the entire synthesis. The synthesis was performed on a 100 μmol scale (using 100 μmol of scaffold **2** and 200 μmol of the Ramage ChemMatrix resin). After the final TFA cleavage, 57 mg of crude compound **8** were obtained. The crude compound was extracted twice with Et_2_O and passed through a C-4 cartridge, as explained for compound **7**, to afford compound **8** (23 mg, pale yellow solid, overall yield from compound **2** of 24%). The purity of compound **8** was verified by RP-HPLC (Method B, Supplementary Figure S6). Crude compound **8** was also analyzed by elemental analysis to check for the presence of residual copper, and ^19^F-NMR was used to quantify the amount of compounds containing the CF_2_ arm present in the crude product (*vide infra*).

Analytical HPLC: (Method B) *t*_R_ = 22.4 min, purity = 71%. ^1^H-NMR (500 MHz; DMSO-*d*_6_; T = 100 °C): δ_H_ 0.85 (3H, t, ^3^*J*_HH_ = 7.0 Hz, –CH_3_), 1.25–1.29 (8H, m, 4 × –CH_2_–), 1.82 (2H, m, –CH_2_–), 2.18 (2H, m, –CH_2_–), 2.26 (2H, m, –CH_2_–CO), 3.94 (2H, t, *J*_HF_ = 14.2 Hz, –CH_2_–N), 4.30 (2H, t, ^3^*J*_HH_ = 7.2, –CH_2_–N), 4.57 (2H, d, ^3^*J*_HH_ = 5.6 Hz, –CH_2_–N), 4.71 (2H, s, –CH_2_–N), 5.64 (2H, s, –CH_2_–N), 7.25 (1H, d, ^3^*J*_HH_ = 7.5 Hz, CH_arom_), 7.35 (1H, tt, ^3^*J*_HH_ = 7.4 Hz, ^4^*J*_HH_ = 1.2 Hz, CH_arom_), 7.44 (3H, m, 3 × CH_arom_), 7.60 (4H, m, 4 × CH_arom_), 7.68 (1H, t, ^3^*J*_HH_ = 7.5 Hz, CH_arom_), 7.76 (1H, d, ^3^*J*_HH_ = 7.5 Hz, CH_arom_), 7.895 (1H, s, CH_arom_), 7.99 (1H, d, ^3^*J*_HH_ = 7.5 Hz, CH_arom_), 8.025 (1H, s, CH_arom_), 8.03 (1H, s, CH_arom_), 8.10 (1H, br s, CH_arom_), 8.11 (1H, br s, CH_arom_), 8.48 (1H, t, ^4^*J*_HH_ = 1.6 Hz, CH_arom_), 8.57 (1H, s, –N=CH), 8.94 (1H, t, ^3^*J*_HH_ = 5.6 Hz, >NH), 11.88 (1H, br s, >NH). ^13^C-NMR (125.8 MHz; DMSO-*d*_6_; T = 100 °C): δ_C_ 13.23 (1C, –CH_3_), 21.42 (1C, –CH_2_–), 25.50 (1C, –CH_2_–), 27.10 (1C, –CH_2_–), 27.55 (1C, –CH_2_–), 29.24 (2C, 2 × –CH_2_–), 30.08 (1C, –CH_2_–), 30.59 (1C, –CH_2_–), 34.90 (1C, –CH_2_–N), 43.90 (1C, –CH_2_–N), 48.73 (1C, –CH_2_–N), 49.06 (1C, –CH_2_–N), 52.75 (1C, –CH_2_–N), 122.32 (1C, CH_arom_), 122.84 (1C, CH_arom_), 123.50 (1C, CH_arom_), 126.00 (1C, CH_arom_), 126.08 (2C, 2 × CH_arom_), 126.32 (2C, 2 × CH_arom_), 126.43 (1C, CH_arom_), 127.18 (1C, CH_arom_), 128.14 (1C, CH_arom_), 128.47 (3C, 3 × CH_arom_), 128.96 (1C, CH_arom_), 129.57 (1C, CH_arom_), 130.57 (1C, CH_arom_), 133.75 (1C, C_arom_), 135.31 (1C, C_arom_), 135.85 (1C, C_arom_), 136.09 (1C, C_arom_), 139.51 (1C, C_arom_), 140.55 (1C, C_arom_), 142.31 (1C, C_arom_), 144.25 (1C, C_arom_), 164.77 (1C, –CO–NH), 165.79 (1C, –CO–NH), 170.24 (1C, –CO–NH) (–CO–N< and four C_arom_ missing). IR (KBr) ν_max_ = 3433 (s, vbr, NH + H_2_O), 1664 (s, br, C=O). HRMS (*m*/*z*): calcd for C_48_H_50_F_5_N_11_NaO_4_^+^ (M + Na)^+^ 962.38596, found 962.38613. Elemental analysis (Cu): 97–103 ppm.

In a parallel experiment, we prepared **8** by the same methodology as described above, but, before the final cleavage, the resin was treated with gaseous H_2_S as described below. We obtained 39 mg of crude compound **8** (41 μmol, 41%, if pure) starting from 82 mg of scaffold **2** (100 μmol).

#### 3.2.7. Compound **8** (High Copper Load)

The solid-phase synthesis of compound **8** was repeated as described above but used a higher load of copper (II) sulfate as well as sodium ascorbate. Thus, for the two CuAAC reactions (to produce resin-bound compounds **6s** and **8s**), the following protocol was applied. The resin was washed with 3 × 5 mL of MeOH and *t*BuOH/water (1:1, *v*/*v*; 1 min each). The following solutions were sequentially added to the resin: (i) azide **9** or **10** (500 μmol, 5.0 eq.) in 1 mL of *t*BuOH/water (1:1, *v*/*v*); (ii) a solution of sodium ascorbate (99 mg, 500 μmol, 5 eq.) in 1 mL of *t*BuOH/water (1:1, *v*/*v*); and (iii) copper(II) sulfate pentahydrate (25 mg, 100 μmol, 1 eq.) in 1 mL of *t*-BuOH/water (1:1, *v*/*v*). After stirring for 16 h at r.t., the coupling was complete. The resin was washed with 3 × 5 mL of *t*BuOH/water (1:1, *v*/*v*), H_2_O, AcOH (to solubilize copper(I) salts), H_2_O, DMF, DCM, MeOH, and DCM (1 min each). The resin was then treated with gaseous hydrogen sulfide (*vide infra*). Before the final cleavage, the resin was washed with 3 × 5 mL DCM and dried over NaOH pellets at 10–100 Pa. The cleavage mixture (75 mg) was extracted twice with Et_2_O, passed through a C-4 cartridge as explained for compound **7**, and lyophilized to afford final compound **8** (44 mg, pale yellow solid, overall yield from compound **2** of 47%). The purity of compound **8** was verified by RP-HPLC (Method B, Figure S7B).

Analytical HPLC: (Method B) purity = 64%. Elemental analysis (Cu): 15 ppm.

#### 3.2.8. Compound **11**

(1) Acylhydrazone formation: The resin-bound compound **5s** (100 μmol of scaffold) was capped with Boc groups as described for compound **7**. The resin was washed with 3 × 5 mL of DCM, DMF, DCM, MeOH, and ACN (1 min each). A solution of 1-butyraldehyde (45 μL, 500 μmol, 5.0 eq.) in 10% TEA/ACN (*v*/*v*, 2.5 mL) was added to the resin. The mixture was stirred at r.t. for 16 h. The resin was then washed with 3 × 5 mL of ACN (1 min each).
(2) Acylhydrazone reduction: The resin containing the acylhydrazone derivative was washed with 5 × 5 mL of dry EtOH. A cooled solution (0 °C) of sodium borohydride (38 mg, 1000 μmol, 10 eq.) in dry EtOH (3 mL) was added to the resin. The syringe was left upside down (to avoid projection) at 5 °C for 30 min and at r.t. for 3 h. The resin was then washed with 3 × 5 mL of MeOH, H_2_O, DMF, DCM, MeOH, and DCM (1 min each) to give resin-bound compound **11s**.

A sample of resin-bound compound **11s** (*ca*. 50% of the total amount) was transferred to a fritted glass micro-reactor and dried under reduced pressure. The product was cleaved using the procedure described for compound **5**. The purity of crude compound **11** (23 mg) was verified by RP-HPLC (Method B, Supplementary Figure S8). Compound **11** was purified using RP-HPLC (Method A) and lyophilized (8.9 mg, yield from scaffold **2** *ca*. 30%, white powder).

Analytical HPLC: (Method B) *t*_R_ = 19.8 min, crude purity = 70%. ^1^H-NMR (500 MHz; DMSO-*d*_6_; T = 100 °C): δ_H_ 0.92 (3H, t, ^3^*J*_HH_ = 7.5 Hz, CH_3_), 1.06 (21H, m, Si(CH(CH_3_)_2_)_3_), 1.40 (2H, m, –CH_2_–), 1.51 (2H, m, –CH_2_–), 2.19 (2H, m, –CH_2_–), 2.28 (2H, m, –CH_2_–CO), 3.15 (1H, t, ^4^*J*_HH_ = 2.4 Hz, –C≡CH), 4.01 (2H, t, ^3^*J*_HF_ = 14.5 Hz, –CH_2_–N), 4.17 (2H, d, ^3^*J*_HH_ = 5.6 Hz, –CH_2_–N), 4.23 (2H, br d, ^4^*J*_HH_ = 2.4 Hz, –CH_2_–N), 7.98 (1H, t, ^3^*J*_HH_ = 1.5 Hz, CH_arom_), 8.00 (1H, t, ^3^*J*_HH_ = 1.5 Hz, CH_arom_), 8.38 (1H, t, ^3^*J*_HH_ = 1.5 Hz, CH_arom_), 8.87 (1H, br t, ^3^*J*_HH_ = 5.6 Hz, >NH) (one –CH_2_–N missing). ^13^C-NMR (125.8 MHz; DMSO-*d*_6_; T = 100 °C): δ_C_ 10.50 (3C, Si(CH(CH_3_)_2_)_3_)), 13.22 (1C, –CH_3_), 18.04 (6C, Si(CH(CH_3_)_2_)_3_), 19.31 (1C, –CH_2_–), 27.08 (1C, –CH_2_–), 29.06 (1C, –CH_2_–), 29.39 (1C, –CH_2_–N), 29.98 (1C, t, ^2^*J*_CF_ = 24.5 Hz, –CH_2_–), 38.50 (1C, –CH_2_–N), 48.80 (1C, –CH_2_–N), 50.35 (1C, –CH_2_–N), 75.14 (1C, ≡C–H), 78.15 (1C, ≡C–), 81.80 (1C, ≡C–), 105.15 (1C, ≡C–), 123.26 (1C, t, ^1^*J*_CF_ = 243.8 Hz, >CF_2_), 127.33 (1C, CH_arom_), 127.43 (1C, CH_arom_), 127.63 (1C, CH_arom_), 133.60 (1C, C_arom_), 134.71 (1C, C_arom_), 135.29 (1C, C_arom_), 164.54 (1C, –CO–NH), 165.62 (1C, –CO–NH), 172.00 (1C, –CO-NH_2_) (–CO–N< missing). HRMS (*m*/*z*): calcd for C_33_H_50_F_2_N_5_O_4_Si^+^ [M + H]^+^ 646.35946, found 646.35970.

#### 3.2.9. Compound **12**

From resin-bound compound **11s**, compound **12** was obtained following the procedures described for compounds **6** and **8** (low copper load). After the final TFA cleavage, 20 mg of crude compound **12** were obtained. The crude compound was extracted twice with Et_2_O and passed through a C-4 cartridge, as explained for compound **7**. The purity of the crude compound **12** was verified by RP-HPLC (Method B, Supplementary Figure S9). Crude compound **12** was then purified using RP-HPLC (Method B) and lyophilized to give pure compound **12** (3.1 mg, white powder).

Analytical HPLC: (Method B) *t*_R_ = 17.9 min. ^1^H-NMR (500 MHz; DMSO-*d*_6_; T = 100 °C): δ_H_ 0.86 (3H, t, ^3^*J*_HH_ = 7.0 Hz, –CH_3_), 1.26 (8H, m, 4 × –CH_2_–), 1.81 (2H, m, –CH_2_–), 2.19 (2H, m, –CH_2_–), 2.27 (2H, m, –CH_2_–CO), 3.91 (2H, t, *J*_HF_ = 14.4 Hz, –CH_2_–N), 4.29 (2H, t, ^3^*J*_HH_ = 7.1, –CH_2_–N), 4.56 (2H, d, ^3^*J*_HH_ = 5.5 Hz, –CH_2_–N), 4.65 (2H, s, –CH_2_–N), 5.64 (2H, s, –CH_2_–N), 7.30 (1H, dm, ^3^*J*_HH_ = 7.5 Hz, CH_arom_), 7.36 (1H, tt, ^3^*J*_HH_ = 7.5 Hz, ^4^*J*_HH_ = 1.2 Hz, CH_arom_), 7.44 (3H, m, 3 × CH_arom_), 7.60 (4H, m, 4 × CH_arom_), 7.86 (1H, s, CH_arom_), 8.02 (1H, s, CH_arom_), 8.05 (1H, t, ^4^*J*_HH_ = 1.6 Hz, CH_arom_), 8.12 (1H, t, ^4^*J*_HH_ = 1.6 Hz, CH_arom_), 8.50 (1H, t, ^4^*J*_HH_ = 1.6 Hz, CH_arom_), 9.02 (1H, t, ^3^*J*_HH_ = 5.5 Hz, >NH), 11.89 (1H, br s, >NH). ^13^C-NMR (125.8 MHz; DMSO-*d*_6_; T = 100 °C): δ_C_ 13.20 (1C, –CH_3_), 21.42 (1C, –CH_2_–), 25.43 (1C, –CH_2_–), 27.11 (1C, t, ^3^*J*_CF_ = 4.1 Hz, –CH_2_–), 27.52 (1C, –CH_2_–), 29.10 (1C, –CH_2_–), 30.06 (1C, t, ^2^*J*_CF_ = 24.3 Hz, –CH_2_–), 30.58 (1C, –CH_2_–), 34.89 (1C, –CH_2_–N), 44.00 (1C, –CH_2_–N), 48.62 (1C, –CH_2_–N), 49.16 (1C, –CH_2_–N), 52.63 (1C, –CH_2_–N), 122.73 (1C, CH_arom_), 122.80 (1C, CH_arom_), 126.07 (1C, CH_arom_), 126.09 (1C, CH_arom_), 126.43 (2C, 2 × CH_arom_), 126.58 (1C, CH_arom_), 127.19 (1C, CH_arom_), 128.48 (2C, 2 × CH_arom_), 128.78 (1C, CH_arom_), 128.90 (1C, CH_arom_), 129.17 (1C, CH_arom_), 129.46 (1C, CH_arom_), 131.33 (1C, C_arom_), 134.89 (1C, C_arom_), 136.09 (1C, C_arom_), 136.34 (1C, C_arom_), 139.53 (1C, C_arom_), 140.54 (1C, C_arom_), 141.84 (1C, C_arom_), 144.73 (1C, C_arom_), 164.60 (1C, –CO–), 165.71 (1C, –CO–), 170.16 (1C, –CO–), 172.01 (1C, –CO–NH_2_). HRMS (*m*/*z*): calcd for C_40_H_44_F_2_N_9_O_5_^−^ 768.34390 [M − H]^−^, found 768.34363; calcd for C_40_H_45_F_2_N_9_NaO_5_^+^ 792.34039 [M + Na]^+^, found 792.34059.

#### 3.2.10. Compound **13**

Compound **13** was synthesized following the procedure described for compound **8** (low copper load), replacing the 3-(trifluoromethyl)benzaldehyde in the synthesis of compound **7** with 4-methoxybenzaldehyde (122 μL, 100 μmol, 10 eq.) and treating the resin with gaseous hydrogen sulfide after each CuAAC. After the final cleavage, the crude product (56 mg) was extracted twice with Et_2_O, passed through a C-4 cartridge as explained for compound **7**, and lyophilized to afford 39 mg (43%) of pale-yellow powder. Part of this powder (20 mg) was purified using RP-HPLC (Method A) and lyophilized (5.4 mg, white powder).

Analytical HPLC: (Method B) *t*_R_ = 20.4 min. ^1^H-NMR (500 MHz; DMSO-*d*_6_; T = 100 °C): δ_H_ 0.85 (3H, t, ^3^*J*_HH_ = 7.5 Hz, CH_3_), 1.24–1.29 (8H, m, 4 × –CH_2_–), 1.82 (2H, m, –CH_2_–), 2.19 (2H, m, –CH_2_–), 2.26 (2H, m, –CH_2_–), 3.83 (3H, s, –OCH_3_), 3.94 (2H, br t, ^3^*J*_HF_ = 14.2 Hz, N–CH_2_–), 4.30 (2H, t, ^3^*J*_HH_ = 7.1 Hz, N–CH_2_–), 4.57 (2H, d, ^3^*J*_HH_ = 5.6 Hz, N–CH_2_–), 4.70 (2H, br s, N–CH_2_–), 5.63 (2H, s, N–CH_2_–), 7.00 (2H, br d, ^3^*J*_HH_ = 8.5 Hz, 2 × CH_arom_), 7.25 (2H, br d, ^3^*J*_HH_ = 7.5 Hz, 2 × CH_arom_), 7.36 (1H, m, CH_arom_), 7.45 (3H, m, 3 × CH_arom_), 7.60 (3H, m, 3 × CH_arom_), 7.65 (2H, br d, ^3^*J*_HH_ = 8.5 Hz, 2 × CH_arom_), 7.89 (1H, s, CH_arom_), 8.03 (1H, m, CH_arom_), 8.08 (2H, br, 2 × CH_arom_), 8.46 (1H, br, CH_arom_), 8.93 (1H, br d, ^3^*J*_HH_ = 5.6 Hz, NH) (–N=CH missing). ^13^C-NMR (125.8 MHz; DMSO-*d*_6_; T = 100 °C): δ_C_ 13.25 (1C, CH_3_), 21.45 (1C, –CH_2_–), 25.52 (1C, –CH_2_–), 27.14 (1C, –CH_2_–), 27.57 (1C, –CH_2_–), 29.27 (1C, –CH_2_–), 30.11 (1C, –CH_2_–), 30.62 (1C, –CH_2_–), 34.93 (1C, –CH_2_–N), 43.94 (1C, –CH_2_–N), 48.66 (1C, –CH_2_–N), 49.08 (1C, –CH_2_–N), 52.77 (1C, –CH_2_–N), 55.14 (1C, OCH_3_), 114.23 (2C, 2 × CH_arom_), 122.33 (1C, CH_arom_), 123.52 (1C, CH_arom_), 126.03 (1C, CH_arom_), 126.11 (1C, CH_arom_), 126.36 (1C, CH_arom_), 126.47 (1C, CH_arom_), 127.22 (1C, CH_arom_), 127.94 (1C, CH_arom_), 128.42 (2C, 2 × CH_arom_), 128.51 (2C, 2 × CH_arom_), 128.99 (3C, 3 × CH_arom_), 134.13 (1C, C_arom_), 134.74 (1C, C_arom_), 135.79 (1C, C_arom_), 136.14 (1C, C_arom_), 139.52 (1C, C_arom_), 140.57 (1C, C_arom_), 142.26 (1C, C_arom_), 144.30 (1C, C_arom_), 160.89 (1C, C_arom_), 164.86 (1C, –CO–NH) (–CO–N<, –CO–NH_2_, –CO–NH and three C_arom_ missing). HRMS (*m*/*z*): calcd for C_48_H_53_F_2_N_11_NaO_5_^+^ 924.40914 [M + Na]^+^, found 924.40973.

### 3.3. Hydrogen Sulfide Treatment

The resin was washed with 3 × 5 mL of ACN and suspended in 5 mL of ACN. Gaseous hydrogen sulfide was aspirated through the syringe, and the syringe was left for 30 min (a dark coloration appears). The resin was then washed with 3 × 5 mL of ACN and DCM.

CAUTION: hydrogen sulfide is a toxic gas that should be only manipulated under a fume hood.

### 3.4. Determination of the Loading of **4** on Resin

(1) Construction of the curve: scaffold **2** (16.5 mg, 20.3 μmol) was dissolved in 40% DCM/ACN (*v*/*v*). This mother solution was diluted 2, 4, 8, 16, and 100 times to obtain five solutions at different concentrations. The absorbance of these solutions at 301 nm (absorption of the Fmoc group) was determined. The experiment was duplicated, and the following equation—linking the absorbance and concentration of scaffold **2**—was obtained: A = 4.34c + 0.0828 (R^2^ = 0.999), where A is the absorbance and c the concentration in μM.
(2) Loading determination: Scaffold **2** (82 mg, 100 μmol) was loaded onto Ramage ChemMatrix^®^ resin (667 mg, 200 μmol) following the protocol described for compound **5**. After the reaction, the resin was washed with 3 × 5 mL of ACN, 3 × 5 mL of DCM, 5 mL of ACN, and 5 mL of DCM (1 min each). The washing solutions were transferred to a 50 mL volumetric flask filled with ACN. The absorbance of this solution at 301 nm was measured (blank = 40% DCM/ACN). The quantity of compound **2** present in the solution (unreacted compound) was determined using the equation A = 4.34c + 0.0828. For instance, an absorbance of 1.67 corresponded to 18.3 μmol of scaffold **2**. Thus, 81.7 μmol (100–18.3 μmol) of compound should be attached to the resin, which corresponds to a loading of 40.8% (= (81.7/200) × 100).

### 3.5. ^19^F-NMR Quantitative Analysis

The ^19^F-NMR analysis was carried out as previously described [4]. Briefly, a precisely weighed sample of dry compound **8** (4.8 mg, 5.1 μmol if pure) was transferred to a NMR tube with DMSO-*d*_6_ (0.4 mL). A known amount of commercial ethyl bromodifluoroacetate (100 μL of a 0.1M solution in DMSO-*d*_6_)—the internal standard—was added to the NMR tube. Three ^19^F signals were present for compound **8** (refer to the ^19^F-NMR spectrum in Supplementary Figure S11): one for the CF_3_ group and two for the CF_2_ group, which result from the slow inter-conversion of conformers [4]. The integration over the two CF_2_^19^F signals, and the comparison to the standard signal, showed a content of fluorine-containing compounds in the sample of 4.8 μmol. These data show that our fluorine label can reliably quantify the content of fluorine-containing scaffold-**8**-related compound(s) by ^19^F-NMR.

## 4. Conclusions

We developed a new variant of a trifunctional scaffold designed for the solid-phase synthesis of combinatorial libraries. The scaffold **2** can be substituted by two different azides and an aromatic aldehyde, which extends its scope compared to our first trialkyne-based scaffold **1**. The derivatization of scaffold **2** with aromatic aldehydes leads, in general, to the desired compounds in sufficient purity for direct biological evaluations. However, electron-rich aromatic hydrazones can be problematic because of their relative instability. Aliphatic aldehydes cannot be used; the resulting aliphatic acylhydrazone is hydrolyzed during cleavage from the resin, and, if reduced to the corresponding aliphatic hydrazide, hydrolysis occurs during the subsequent CuAAC.

We established a one-pot Fmoc-deprotection/acylhydrazone formation protocol that is effective with aromatic aldehydes as well as with aliphatic aldehydes. This methodology is based on the use of TEA as the base for the Fmoc-deprotection. Because Fmoc-hydrazine is easily introduced onto carboxylic acids (through simple amide coupling), this methodology could be of broad use. We solved the problem of the accumulation of interfering copper ions in the resin: treatment of the resin with gaseous hydrogen sulfide traps the copper ions as inert copper sulfide. This protocol is easy to implement and permits the use of one equivalent of copper during the CuAACs.

Overall, scaffold **2** offers a new chemical space for the development of bioactive compounds, and the information provided here will be helpful to chemists working on multifunctional scaffolds or performing solid-phase CuAACs.

## Supplementary Materials

Experimental for azido compounds **9** and **10** (Scheme S1), study of the copper-promoted hydrolysis of acylhydrazide in solution (Figure S1), study of the acylhydrazone formation in the presence of copper(II) ions (Figure S2 and Table S1), study of the hydrolysis of **13** in the presence of copper(II) ions (Table S2, Scheme S5), discussion of the step order (Scheme S6 and Figure S3), RP-HPLC chromatograms for compounds **2**, **7**, **8** (low and high copper load), **11**, **12**, and **13** (Figures S4–S10), and ^19^F-NMR spectra for the quantitative analysis of compound **8** (Figures S11). Supplementary materials can be accessed at: http://www.mdpi.com/1420-3049/20/10/19310/s1.
