# Supplementary material for: A CuAAC–Hydrazone–CuAAC Trifunctional Scaffold for the Solid-Phase Synthesis of Trimodal Compounds: Possibilities and Limitations
Source: Molecules. 2015 Oct 23;20(10):19310–29. doi: 10.3390/molecules201019310 (PMC6332392; doi:10.3390/molecules201019310)
Supplement: Supplementary file 1 [file molecules-20-19310-s001.pdf]

Supplementary Material

A CuAAC-Hydrazone-CuAAC Trifunctional Scaffold for the Solid-Phase Synthesis of Trimodal Compounds: Possibilities and Limitations

Benjamin Fabre, Jan Pícha, Václav Vaněk, Miloš Buděšínský and Jiří Jiráček *

Institute of Organic Chemistry and Biochemistry, Academy of Sciences of the Czech Republic, v.v.i., Flemingovo n. 2, 16610 Praha 6, Czech Republic.

Synthesis of azides **9** and **10** . . . . Page S2
Copper-promoted hydrolysis of hydrazide in solution. . . . . Page S3
➢ Figure S1 . . . . Page S4
Condensation of benzhydrazide and 3-(trifluoromethyl)benzaldehydes in the presence of copper(II) ions . . . . Page S5
➢ Figure S2 . . . . Page S5
➢ Table S1 . . . . Page S5
Hydrolysis of **13** in the presence of copper(II) ions . . . . Page S6
➢ Table S2 . . . . Page S6
➢ Scheme S5 . . . . Page S7
Discussion on the step order of the click reactions. . . . . Page S7
➢ Scheme S6. . . . . Page S7
➢ Figure S3 . . . . Page S8
HPLC chromatograms . . . . Page S9
➢ Figure S4. . . . . Page S9
➢ Figure S5. . . . . Page S9
➢ Figure S6. . . . . Page S10
➢ Figure S7. . . . . Page S10
➢ Figure S8. . . . . Page S11
➢ Figure S9. . . . . Page S11
➢ Figure S10 . . . . Page S12
^{19}F-NMR spectrum of compound **8** (Figure S11) . . . . Page S13
References . . . . Page S13

Synthesis of Azides 9 and 10

Azides were synthesized via classical nucleophilic substitution [1], as shown in Scheme S1. The advancement of the azide formation was monitored by TLC [2].

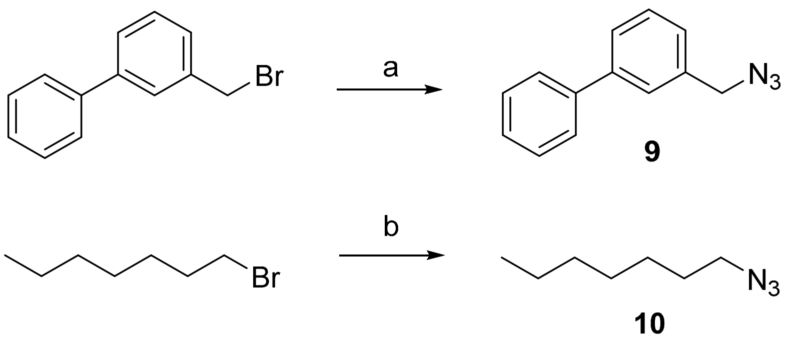

Scheme S1. Synthesis of azides **9** and **10**. (**a**) NaN_3, DMSO, 80 °C overnight (90%); (**b**) NaN_3, DMSO, 80 °C overnight (77%).

3-Phenylbenzylazide 9

A suspension of 3-phenylbenzylbromide (5 g; 20.2 mmol) and NaN_3 (2.6 g; 40.4 mmol) in DMSO (50 mL) was stirred overnight at 80 °C. After cooling to r.t., water (50 mL) was added and the resulting mixture was extracted with diethyl ether (4 × 50 mL). The organic layers were combined, washed with water (2 × 50 mL) and brine (2 × 50 mL), dried over anhydrous sodium sulfate and filtered. The diethyl ether was carefully evaporated under reduced pressure (azide is partially volatile and could be explosive) to give bright brown crude product, which was purified by flash chromatography on silica gel using a linear gradient of diethyl ether in petroleum ether to give **9** (3.8 g, 90%, colorless liquid).

R_f = 0.89 (toluen). ^{1}H-NMR (600 MHz; $CDCl_3$): δ_H 4.39 (2H, s, $–CH_2–N_3$), 7.29 (1H, m, CH_{arom}), 7.36 (1H, m, CH_{arom}), 7.44 (2H, m, 2 × CH_{arom}), 7.45 (1H, m, CH_{arom}), 7.52 (1H, m, CH_{arom}), 7.56 (1H, m, CH_{arom}), 7.59 (2H, m, 2 × CH_{arom}). ^{13}C-NMR (125.8 MHz; $CDCl_3$): δ_C 54.82 (1C, $–CH_2–N_3$), 126.94 (1C, CH_{arom}), 126.97 (1C, CH_{arom}), 127.10 (1C, CH_{arom}), 127.17 (2C, 2 × CH_{arom}), 127.53 (1C, CH_{arom}), 128.80 (2C, 2 × CH_{arom}), 129.25 (1C, CH_{arom}), 135.90 (1C, C_{arom}), 140.61 (1C, C_{arom}), 141.90 (1C, C_{arom}). IR (CCl_4) ν_{max} cm^{-1} 2100 vvs (N_3); 2927 w, 1422 m (CH_2); 3085 w, 3064 m, 3034 m, 1590 w, 1480 m, 1455 m, 699 s (ring). HRMS (CI) calc for $C_{13}H_{11}N_3$ $[M + H]^+$ 209.0953, found: 209.0954.

1-Azidoheptane 10

1-azidoheptane **10** was prepared from 1-bromoheptane (10 g; 55.8 mmol) and NaN_3 (7.3 g; 111.6 mmol), following the method described for azide **9**. After flash chromatography (silica gel, linear gradient diethyl ether in petroleum ether) **10** (6 g, 77%) was obtained as a colorless liquid.

R_f = 0.62 (petroleum ether). ^{1}H-NMR (600 MHz; $CDCl_3$): δ_H 0.89 (3H, t, $^3J_{HH}$ = 7.1 Hz, $–CH_3$), 1.28 (2H, m, $–CH_2–$), 1.30 (2H, m, $–CH_2–$), 1.32 (2H, m, $–CH_2–$), 1.36 (2H, m, $–CH_2–$), 1.60 (2H, m, $–CH_2–$), 3.25 (2H, t, J_{HH} = 7.0 Hz, $–CH_2–N_3$). ^{13}C-NMR (150.9 MHz; $CDCl_3$): δ_C 13.96 (1C, $–CH_3$), 22.50 (1C, $–CH_2–$), 26.62 (1C, $–CH_2–$), 28.77 (1C, $–CH_2–$), 28.79 (1C, $–CH_2–$), 31.63 (1C, $–CH_2–$), 51.42 (1C,

–CH_2–N_3). IR (film) ν_{max} cm^{-1} 2096 vvs (N_3); 2958 s, 2872 s, 1378 w (CH_3); 2930 vs, 2859 s, 1467 m (CH_2). HRMS (CI) calc for $C_7H_{16}N_3$ $[M + H]^+$ 142.1344, found: 142.1346.

Copper-Promoted Hydrolysis of Hydrazide in Solution

We investigated the ability of copper ions to promote the apparent hydrolysis of substituted hydrazides in solution using the model *N'*-butylbenzohydrazide (**14**). The reaction conditions (R1-R3) are shown in Scheme S2.

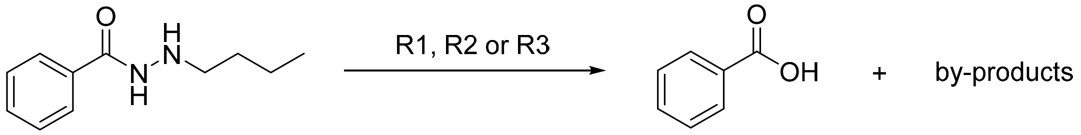

14

Scheme S2. Hydrolysis of **14** in solution. Compound **14** (10 μmol) was dissolved in ACN/H_2O (1:1, *v*/*v*, 1 mL) (R1), or in ACN/H_2O (1:1, *v*/*v*, 1 mL) with 10 μmol $CuSO_4 \cdot 5H_2O$ (R2) or in ACN/H_2O (1:1, *v*/*v*, 1 mL) with 10 μM $CuSO_4 \cdot 5H_2O$ and 50 μmol sodium ascorbate (R3). The solutions were stirred at r.t. in an open vessels.

Three different aqueous solutions of substituted hydrazide **14** (10 μM) without copper (R1), with Cu(II) ions (R2) and with Cu(I) ions (after the reduction with sodium ascorbate, R3) were prepared (Scheme S2). The hydrolysis of compound **14** was followed by HPLC; an aliquot of each solution (10 μL) was taken (at 30 min, 60 min, 210 min and 360 min) and directly injected into HPLC. The hydrolysis of compound **14** is characterized by the appearance of the peak of benzoic acid at 19.272 min (Figure S1). The peaks of benzoic acid and compound **14** were identified by the injections of benzoic acid and compound **14** alone. The peak at 11.576 min could not be identified but may correspond to a complex formed by compound **14** and copper ions. In Figure S1, the HPLC chromatogram of the solution containing Cu(II) ions ($CuSO_4 \cdot 5H_2O$ alone, R2) after 30 min of reaction is shown. The rate of appearance of benzoic acid in the solutions containing copper ions is also shown in Figure S1.

Compound **14** in the solution without Cu ions (R1) remained unchanged after 6 h of the reaction. In contrary, the formation of benzoic acid is observed after 30 min of reaction in the solution with Cu(II) ions (R2). Thus, the presence of Cu(II) indeed promotes the hydrolysis of compound **14** in aqueous media. In the case of the solution with Cu(I) ions ($CuSO_4 \cdot 5H_2O$ and sodium ascorbate, R3), the hydrolysis is much slower (11% of benzoic acid after 6 h at r.t.) and may be attributed to the minor concentration of Cu(II) ions.

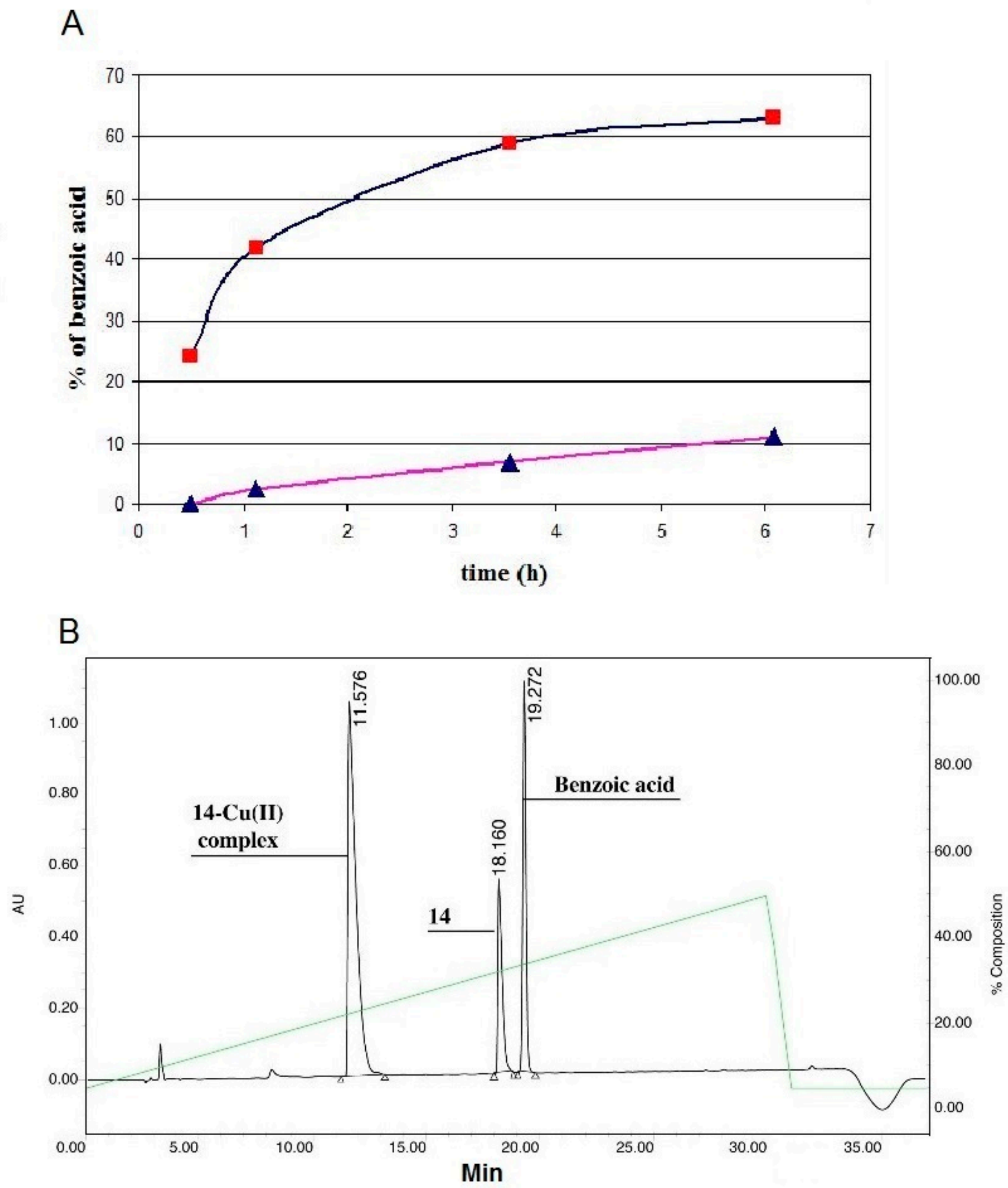

Figure S1. Hydrolysis of **14**. (**A**) Kinetics of the appearance of benzoic acid in the solutions containing Cu(I) (R3, line in magenta, triangles) or Cu(II) ions (R2, line in blue, squares); (**B**) RP-HPLC chromatogram 30 min after the addition of the $CuSO_4{\cdot}5H_2O$ to pure compound **14**. Compounds were detected at 218 nm. The green line reflects the composition of a gradient (% of solvent B). RP-HPLC were carried out using the same system and the same analytical C-8 column as described in the main manuscript. Solvent A: 0.1% TFA (*v*/*v*) in H_2O. Solvent B: 0.1% TFA (*v*/*v*) in ACN.

Condensation of Benzhydrazide and 3-(Trifluoromethyl)benzaldehyde in the Presence of Copper(II) Ions

We investigated if copper(II) ions interfere with the condensation between benzhydrazide and 3-(trifluoromethyl)benzaldehyde. The reaction conditions (R1–R3) are shown in Scheme S3.

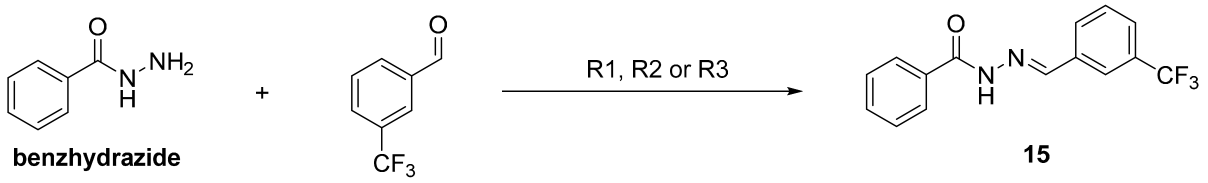

Scheme S3. Condensation reaction in the presence of copper(II) ions. Benzhydrazide (37 µmol) was dissolved in ACN (1 mL) alone (R1), with 1 equivalent of $CuSO_4{\cdot}5H_2O$ (R2) or with 1 equivalent of $CuSO_4{\cdot}5H_2O$ and 5 equivalents of 18-crown-6 (R3). The solutions were stirred at r.t.

The condensation was followed by HPLC; an aliquot of each solution (5 µL) was taken after 210 min of reaction (time necessary for the completion of the reaction without copper) and directly injected into HPLC. As $CuSO_4{\cdot}5H_2O$ has a limited solubility in ACN, 18-crown-6 ether was added in a third reaction (condition R3). The condensation is characterized by the appearance of the peak of **15** at 23.874 min and the disappearance of the peak of benzhydrazide at 7.896 min (Figure S2). As shown in Table S1, the presence of one equivalent of copper has a negligible impact on the reaction advancement.

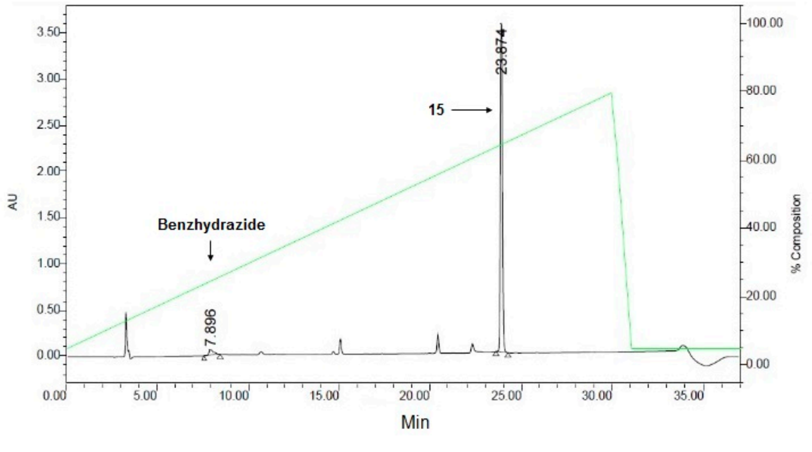

Figure S2. RP-HPLC chromatogram of the reaction R3 after 90 min. The green line reflects the composition of a gradient (% of solvent B). RP-HPLC were carried out using the same system, analytical column and eluents as described in the main manuscript.

Table S1. Advancement of the reactions R1, R2 and R3 after 90 min. The completion of the reaction is calculated by integration of the signals of **15** and benzaldehyde at 218 nm.

Conditions	$CuSO_4{\cdot}5H_2O$	18-Crown-6	Completion
R1	-	-	100%
R2	1 eq.	-	≥99%
R3	1 eq.	5 eq.	96%

Hydrolysis of 13 in the Presence of Copper(II) Ions

We investigated the stability of **13** in the HPLC buffer (50% ACN/water + 0.1% TFA, pH ≈ 2) or in the HPLC buffer with copper(II) ions. The reaction conditions (R1–R2) are shown in Scheme S4.

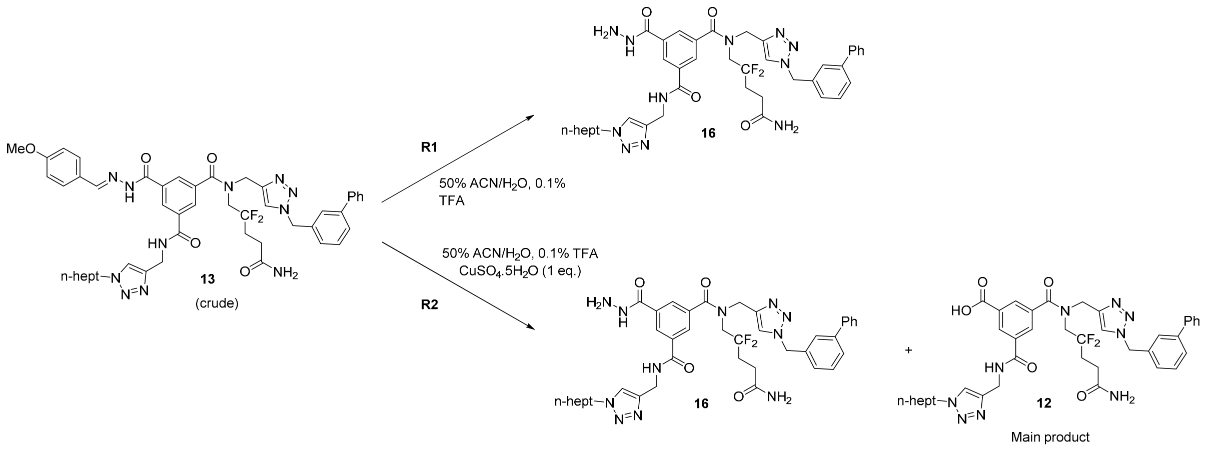

Scheme S4. Hydrolysis of **13** in aqueous media. A solution of crude **13** (1.1 mM) in 50% ACN/water + 0.1% TFA (R1) or in 50% ACN/water + 0.1% TFA with 1 equivalent of $CuSO_4 \cdot 5H_2O$ (R2) was stirred at r.t.

The reactions were followed by HPLC; after 1h of reaction, an aliquot of each solution (10 µL) was directly injected into HPLC. The proportions of compounds **12**, **13** and **16** in the starting crude **13** and after 1 h of reaction (condition R1 or R2) are given in Table S2.

Table S2. Proportion of **12**, **13** and **16** in the starting crude **13** and in reaction mixtures R1 and R2 (after 1 h). The proportions were calculated by integration of signals (peaks) in HPLC chromatograms at 218 nm. The real proportion of **16** in the crude compound **13** is lower than observed by HPLC as hydrolysis of **13** occurred during the HPLC analysis (**16** was not detected in the mass spectrum of the crude compound **13**).

Conditions	% 12	% 13	% 16
Crude **13**	24	57	19
R1	24	16	60
R2	50	40	10

From these results we concluded that the presence of copper ions involves an additional hydrolysis pathway to the classical [3,4] hydrazone hydrolysis. Based on the literature [5–7] we proposed the pathway for the formation of **12** shown in Scheme S5: after hydrolysis of the hydrazone moiety in aqueous acidic medium, the copper-promoted oxidation of the hydrazide **16** would lead to the benzoic acid **12** (after the addition of a molecule of water).

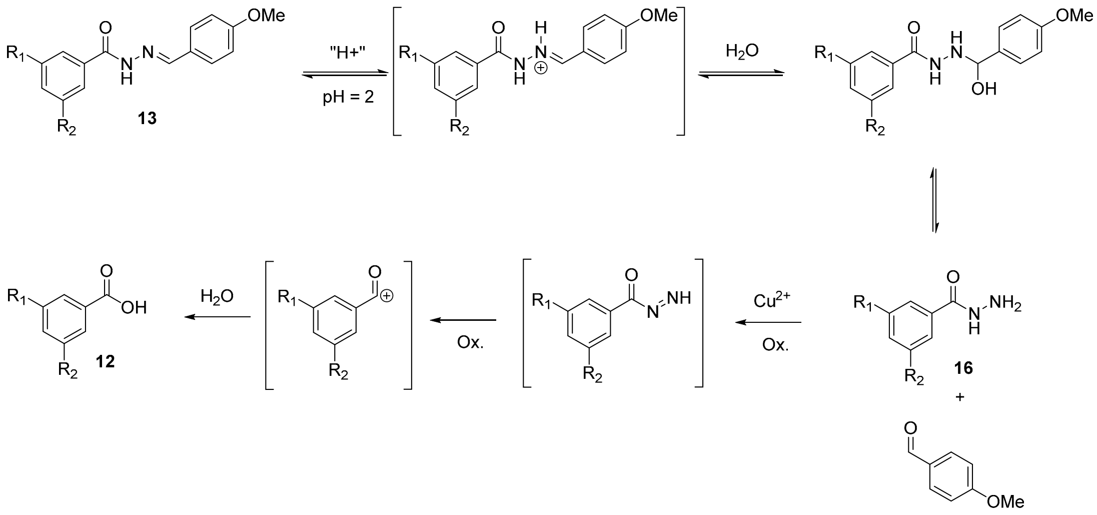

Scheme S5. Proposed pathway for the hydrolysis of **13** in the presence of copper(II) ions.

Discussion on the Step Order of the Click Reactions

We determined the optimal step order for the two first click reactions (CuAAC and hydrazone formation) on solid-phase. The two possible pathways are shown in Scheme S6.

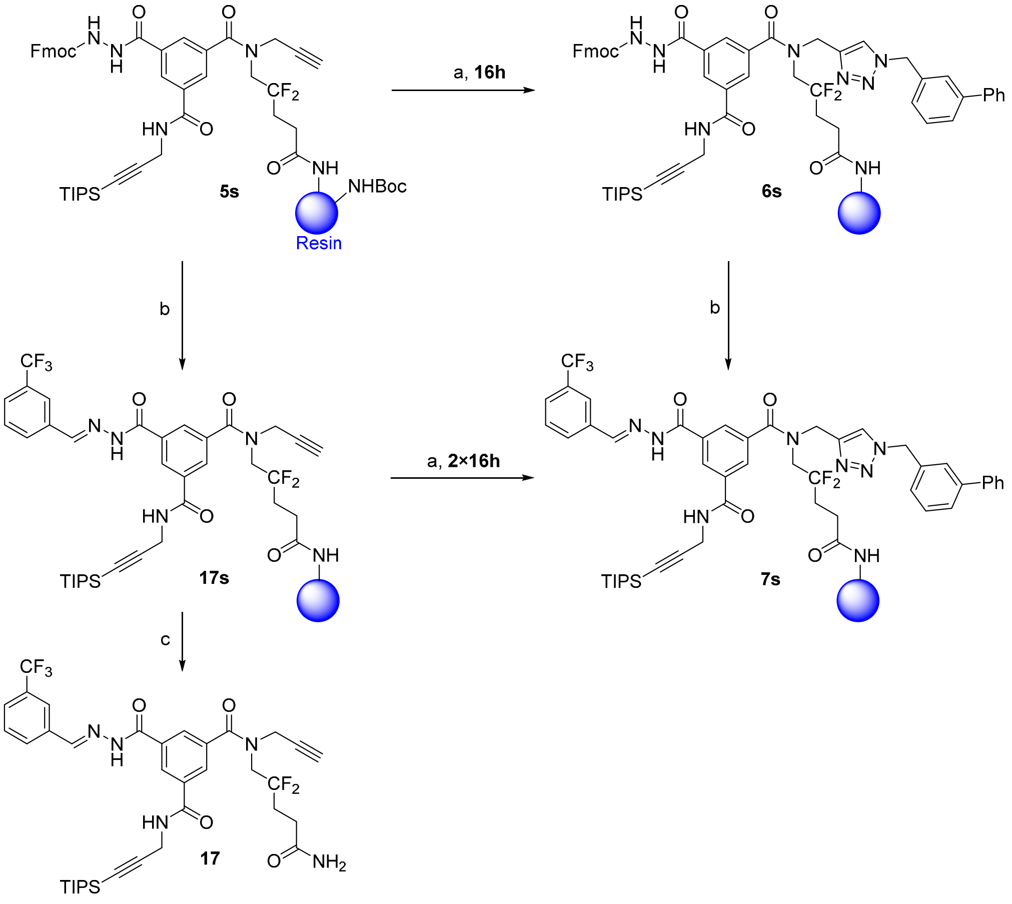

Scheme S6. The two pathways for the synthesis of **7s**. Reaction conditions: (**a**) azide **9** (5 eq.), sodium ascorbate (0.5 eq.), $CuSO_4{\cdot}5H_2O$ (0.1 eq.), tBuOH/water (1:1), r.t.; (**b**) 3-(trifluoromethyl) benzaldehyde (5 eq.), 10% TEA/ACN (*v*/*v*), r.t., 16 h; (**c**) 5% TFA/DCM (*v*/*v*), r.t., 2 × 30 min.

In the pathway proposed in the main manuscript (steps a then b) the CuAAC went to completion after 16 h. In contrast, when the acylhydrazone formation was performed first (step b then step a), the CuAAC was not complete after two treatments of 16 h each: RP-HPLC analysis showed 27% of starting material **17** remaining after the second treatment (Figure S3).

To explain these results, we speculate that the presence of the Fmoc group in the resin-bound compound **6s** impacts the resin properties (lipophilicity, three-dimensional arrangement) and helps the diffusion of the bulky and lipophilic azide **9** into the resin.

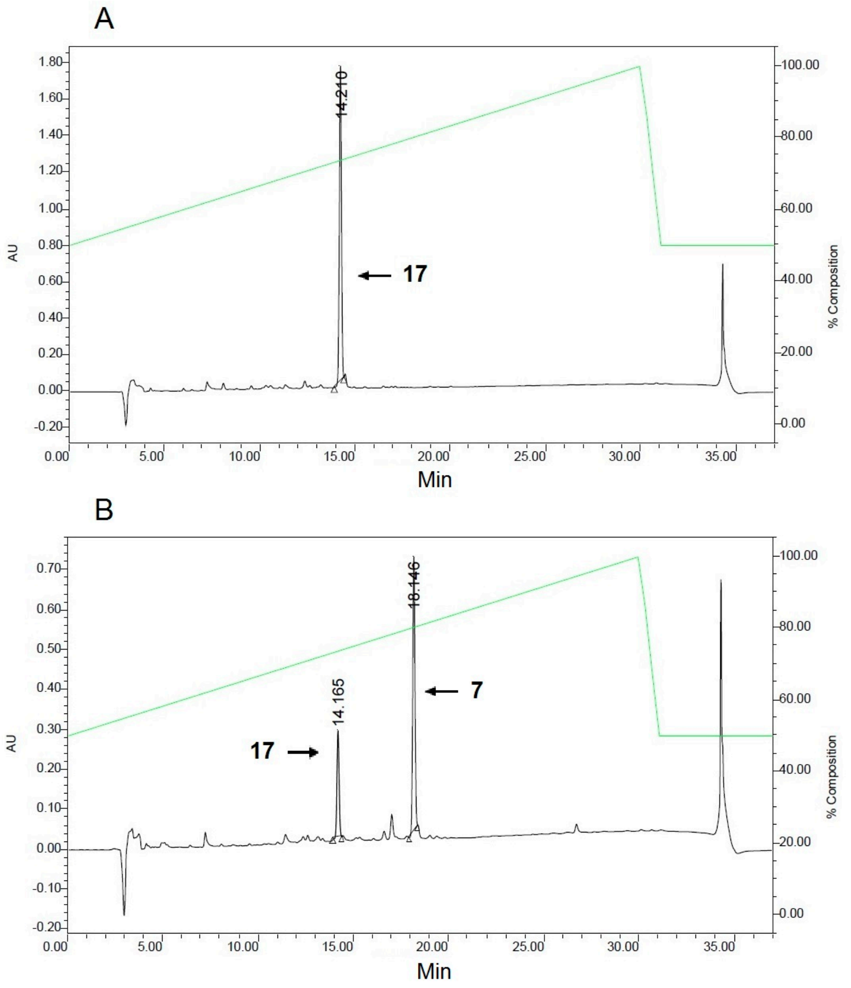

Figure S3. Following of the pathway b then a by RP-HPLC. (**A**) Step b: Compound **17** obtained from **5s** after 16 h of reaction at room temperature; (**B**) Step a: compound **7** obtained from **17s** after 2 × 16 h of reaction at room temperature. A nucleosil 120-5 C18 column (250/4) was used here and the compounds were detected at 218 nm. The green line reflects the composition of the gradient (% of solvent B, Method A).

HPLC Chromatograms

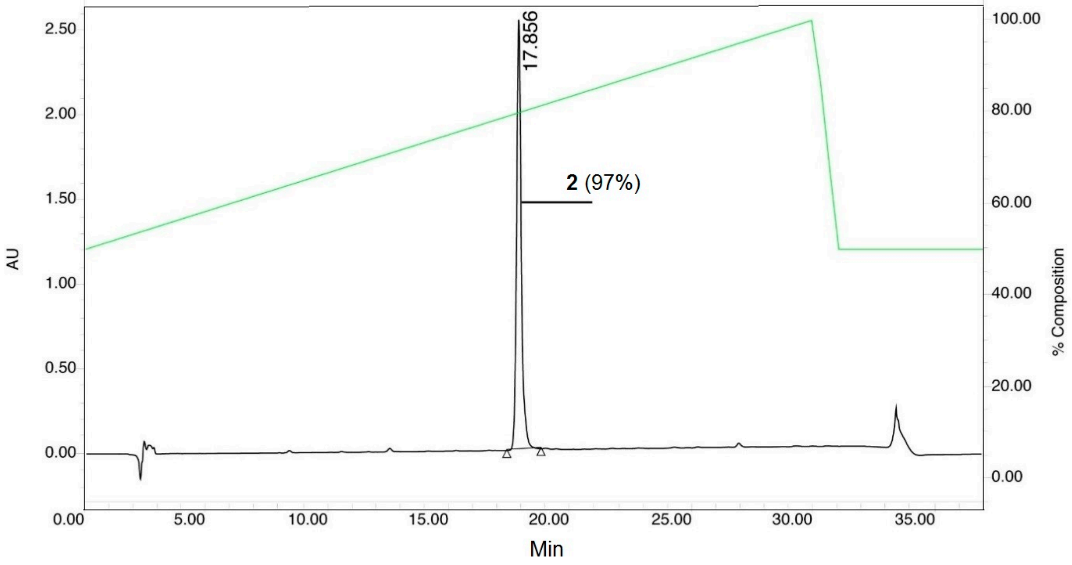

Figure S4. RP-HPLC analysis of compound **2**. Compounds were detected at 218 nm. The green line reflects the composition of the gradient (% of solvent B, Method A). The purity of the compound after the integration of the whole chromatogram at 218 nm is indicated.

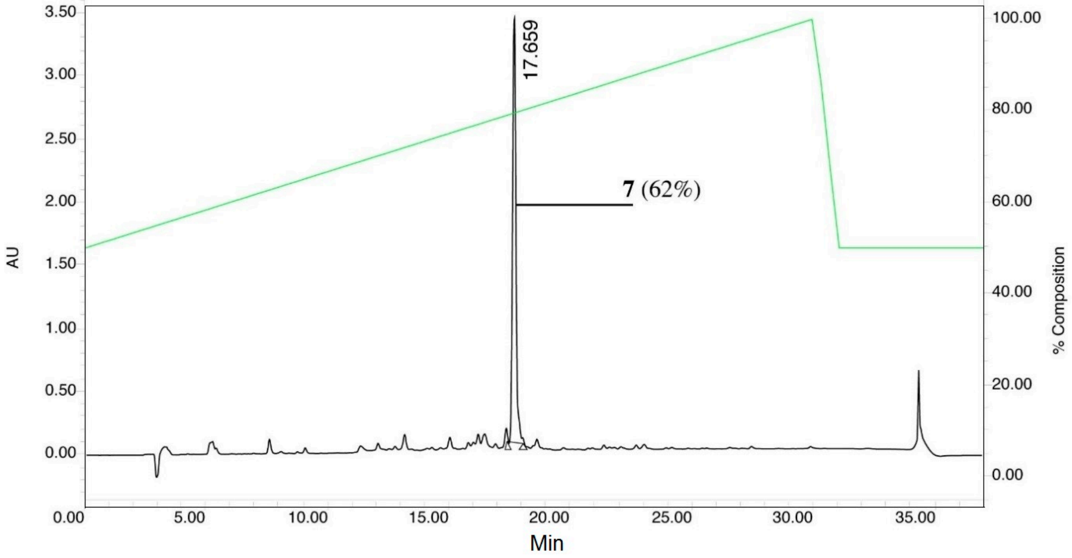

Figure S5. RP-HPLC analysis of compound **7**. Compounds were detected at 218 nm. The green line reflects the composition of the gradient (% of solvent B, Method A). The purity of the compound after integration of the whole chromatogram at 218 nm is indicated.

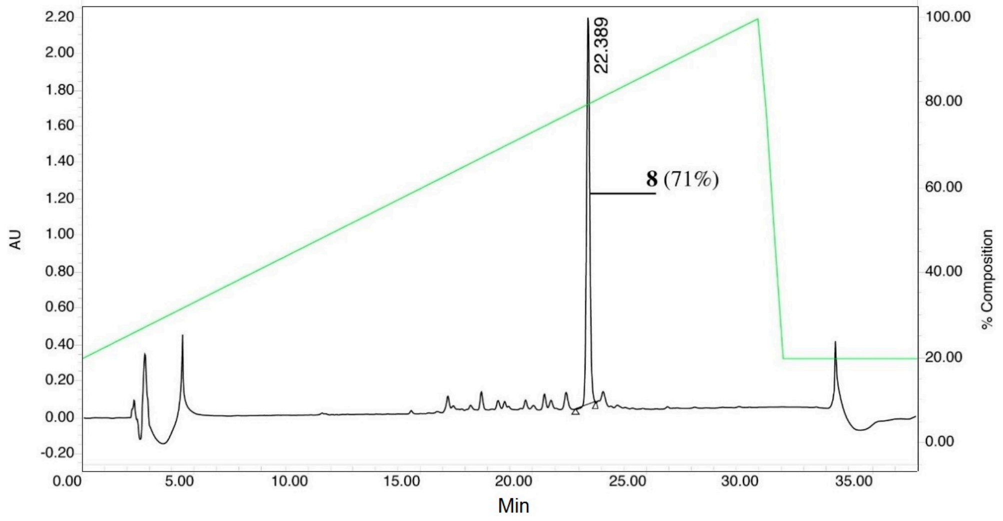

Figure S6. RP-HPLC analysis of compound **8**. Compounds were detected at 218 nm. The green line reflects the composition of the gradient (% of solvent B, Method B). The purity of the compound after the integration of the whole chromatogram at 218 nm is indicated.

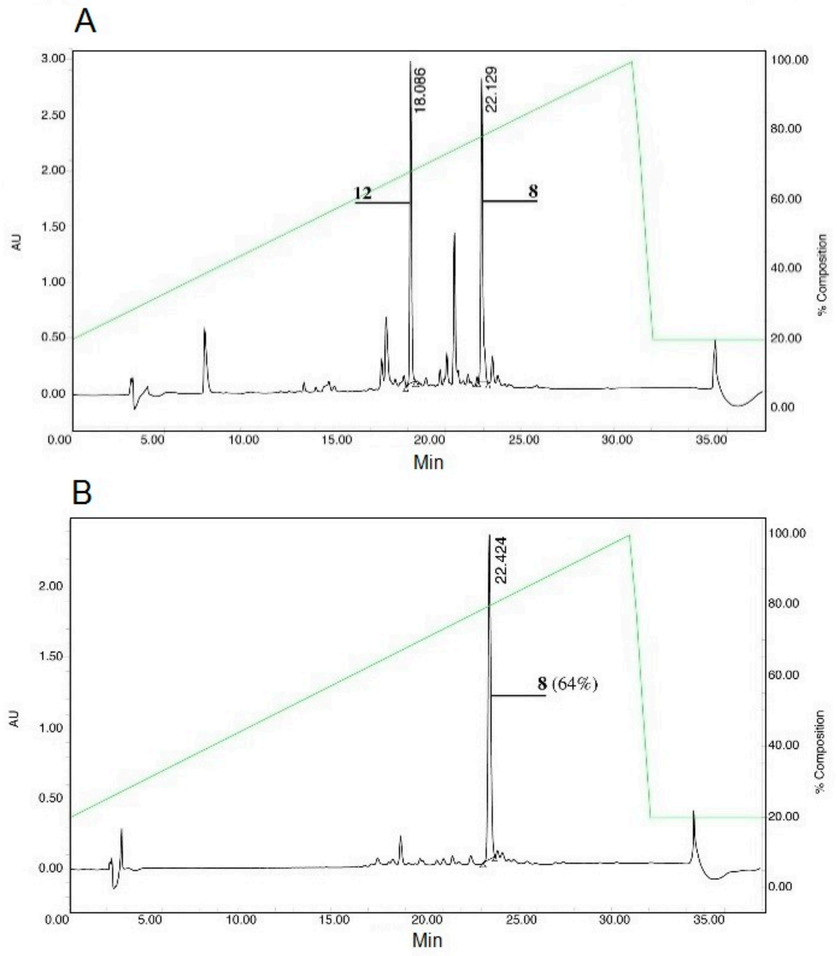

Figure S7. RP-HPLC analysis of the final cleavage mixtures during the synthesis of **8** with a high copper load. (**A**) Without treatments with hydrogen sulfide; (**B**) With treatments of the resin, after each CuAAC, with hydrogen sulfide. Compounds were detected at 218 nm. The green line reflects the composition of the gradient (% of solvent B, Method B).

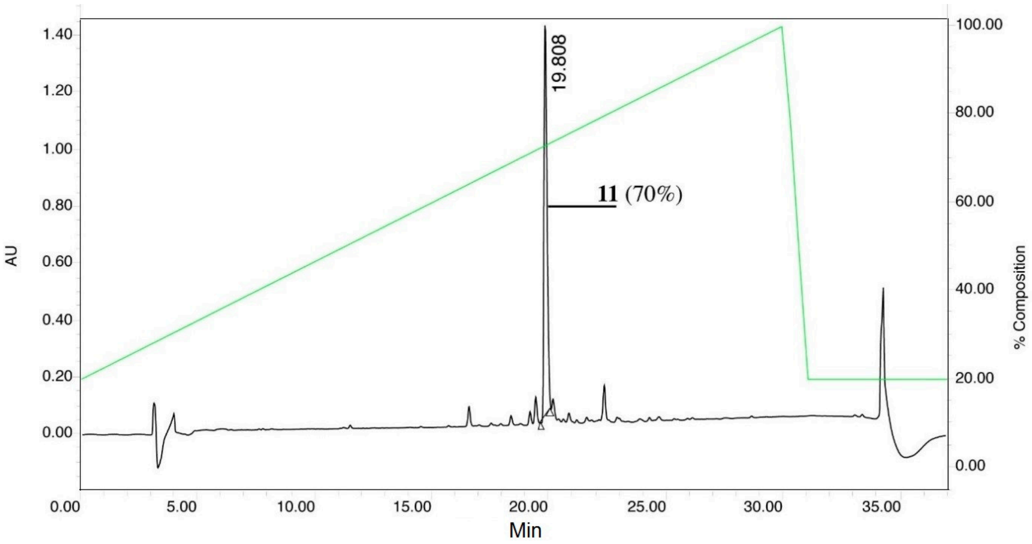

Figure S8. RP-HPLC analysis of compound **11**. Compounds were detected at 218 nm. The green line reflects the composition of the gradient (% of solvent B, Method B). The purity of the compound after the integration of the whole chromatogram at 218 nm is indicated.

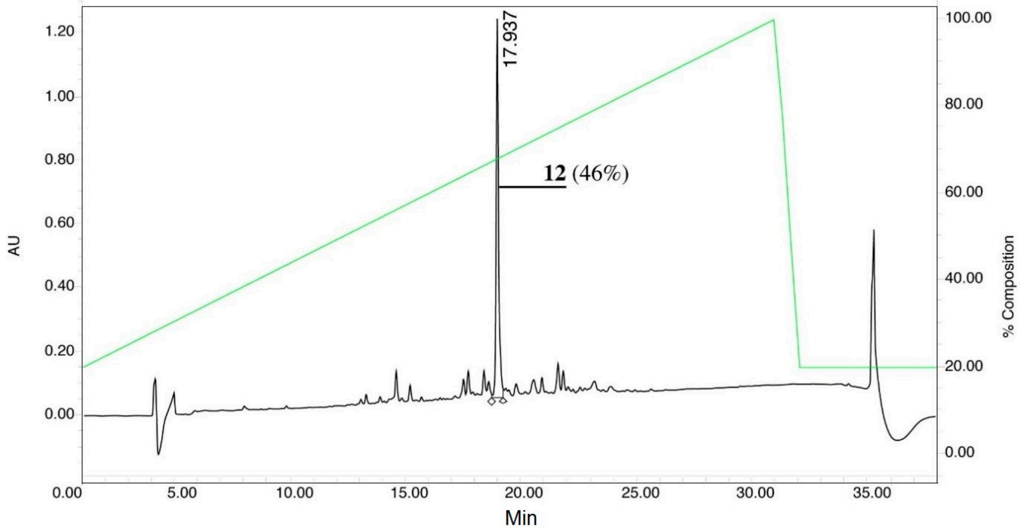

Figure S9. RP-HPLC analysis of compound **12**. Compounds were detected at 218 nm. The green line reflects the composition of the gradient (% of solvent B, Method B). The purity of the compound after the integration of the whole chromatogram at 218 nm is indicated.

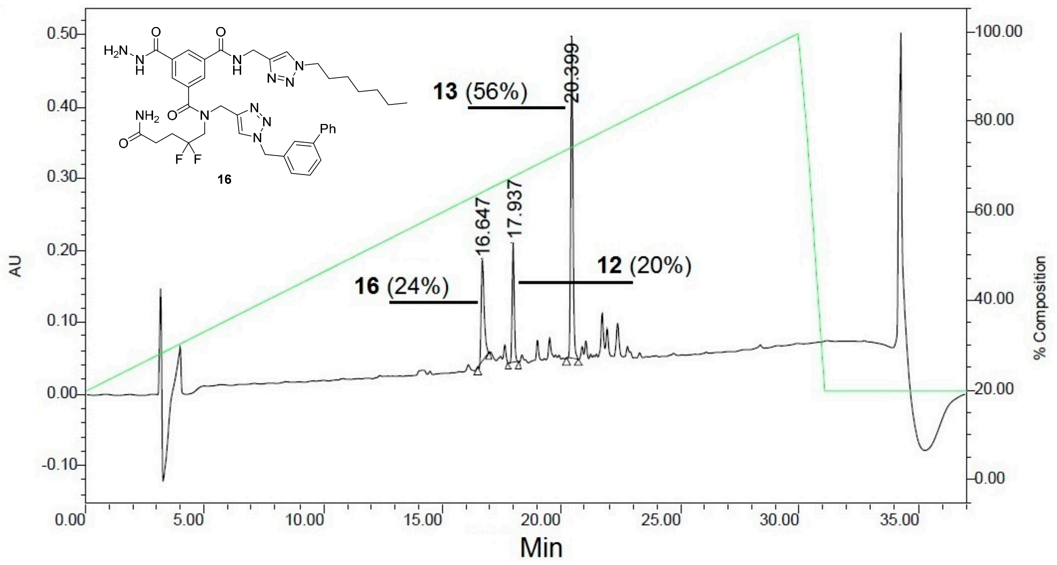

Figure S10. RP-HPLC analysis of compound **13**. Compounds were detected at 218 nm. The green line reflects the composition of the gradient (% of solvent B, Method B). The relative proportions (in %) of the three main peaks (areas at 218 nm) are indicated. As discuss above (Table S2), the hydrazide **16** probably results from the hydrolysis of acylhydrazone **13** in the HPLC buffer.

^{19}F-NMR Spectrum of Compound 8

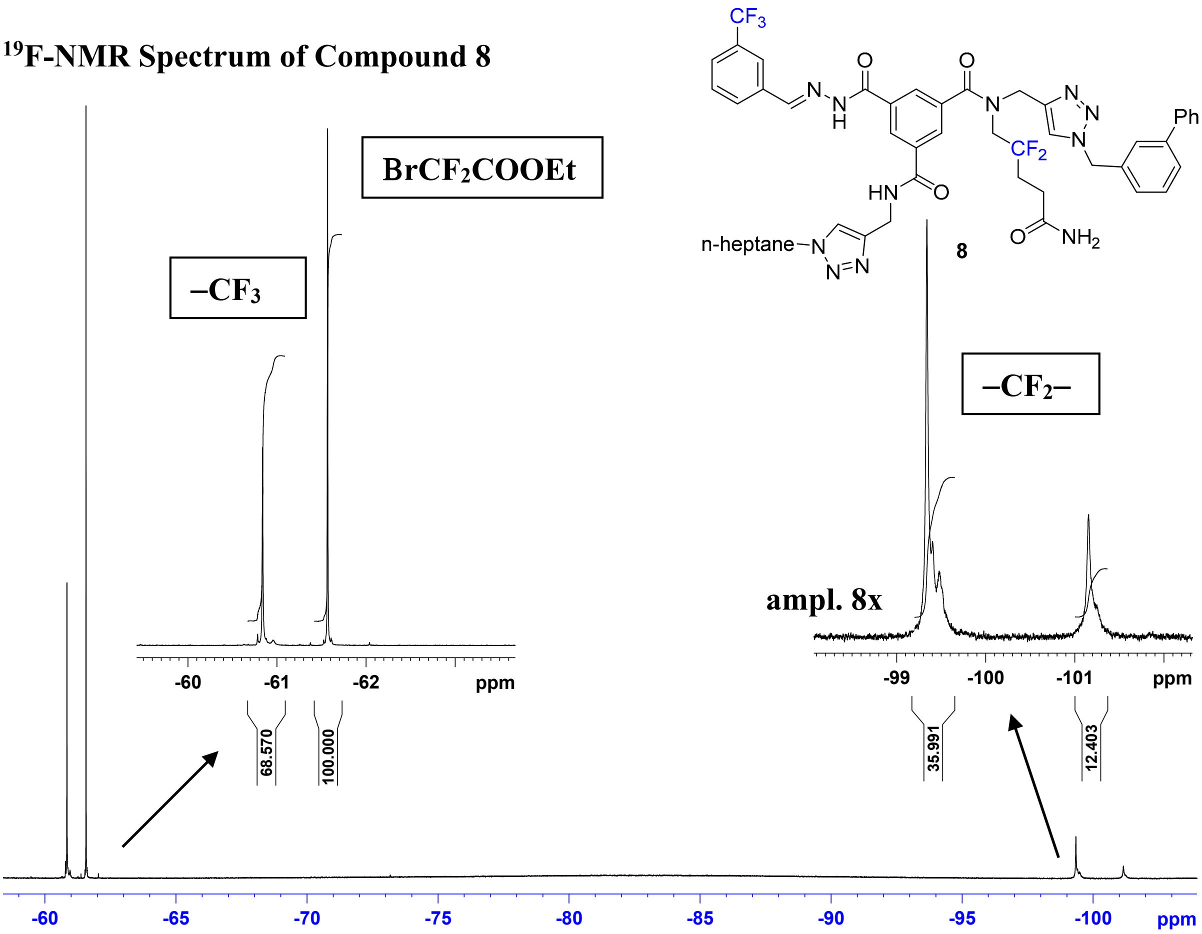

Figure S11. ^{19}F-NMR spectrum of compound **8** with the internal standard $BrCF_2COOEt$ (in DMSO-d_6). The two ^{19}F signals of the CF_2 group result from a slow interconversion of conformers. This has already been proven in our previous work by temperature dependence of the ^{19}F-NMR spectra and the coalescence of signals at ~100 °C [8].

References

1. Haase, J. Large-scale preparation and usage of azides. In *Organic Azides: Syntheses and Applications*, 1st ed.; Bräse, S.; Banert, K.; Eds.; John Wiley & Sons, Ltd.: Chichester, UK, 2010; Volume 2, pp. 29–51.
2. Cegielska, B.; Kacprzak, K.M. Simple and convenient protocol for staining of organic azides on TLC plates by ninhydrin. A new application of an old reagent. *Chem. Anal.* **2009**, *54*, 807–812.
3. Cordes, E.H.; Jencks, W.P. On the Mechanism of Schiff Base Formation and Hydrolysis. *J. Am. Chem. Soc.* **1962**, *84*, 832–837.
4. Cordes, E.H.; Jencks, W.P. The Mechanism of Hydrolysis of Schiff Bases Derived from Aliphatic Amines. *J. Am. Chem. Soc.* **1963**, *85*, 2843–2848.
5. Kumar, M.; Kumar, N.; Bhalla, V.; Sharma, P.R.; Kaur, T. Highly Selective Fluorescence Turn-on Chemodosimeter Based on Rhodamine for Nanomolar Detection of Copper Ions. *Org. Lett.* **2012**, *14*, 406–409.

6. Amos, R.I.J.; Gourlay, B.S.; Schiesser, C.H.; Smith, J.A.; Yates, B.F. A. mechanistic study on the oxidation of hydrazides: application to the tuberculosis drug isoniazid. *Chem. Commun.* **2008**, *14*, 1695–1697.
7. Amos, R. I.J; Gourlay, B.S.; Yates, B.F.; Schiesser, C.H.; Lewis, T.W.; Smith, J.A. Mechanistic investigation of the oxidation of hydrazides: implications for the activation of the TB drug isoniazid. *Org. Biomol. Chem.* **2013**, *11*, 170–176.
8. Vaněk, V.; Pícha, J.; Fabre, B.; Buděšínský, M.; Lepšík, M.; Jiráček, J. The Development of a Versatile Trifunctional Scaffold for Biological Applications. *Eur. J. Org. Chem.* **2015**, *17*, 3689–3701.
